# Spike sorting based on shape, phase, and distribution features, and *K*-TOPS clustering with validity and error indices

**DOI:** 10.1038/s41598-018-35491-4

**Published:** 2018-12-12

**Authors:** Carmen Rocío Caro-Martín, José M. Delgado-García, Agnès Gruart, R. Sánchez-Campusano

**Affiliations:** Division of Neurosciences, Pablo de Olavide University, Seville, 41013 Spain

## Abstract

Spike sorting is one of the most important data analysis problems in neurophysiology. The precision in all steps of the spike-sorting procedure critically affects the accuracy of all subsequent analyses. After data preprocessing and spike detection have been carried out properly, both feature extraction and spike clustering are the most critical subsequent steps of the spike-sorting procedure. The proposed spike sorting approach comprised a new feature extraction method based on shape, phase, and distribution features of each spike (hereinafter SS-SPDF method), which reveal significant information of the neural events under study. In addition, we applied an efficient clustering algorithm based on *K*-means and template optimization in phase space (hereinafter *K*-TOPS) that included two integrative clustering measures (validity and error indices) to verify the cohesion-dispersion among spike events during classification and the misclassification of clustering, respectively. The proposed method/algorithm was tested on both simulated data and real neural recordings. The results obtained for these datasets suggest that our spike sorting approach provides an efficient way for sorting both single-unit spikes and overlapping waveforms. By analyzing raw extracellular recordings collected from the rostral-medial prefrontal cortex (rmPFC) of behaving rabbits during classical eyeblink conditioning, we have demonstrated that the present method/algorithm performs better at classifying spikes and neurons and at assessing their modulating properties than other methods currently used in neurophysiology.

## Introduction

Spike-sorting methods have received intensive attention in neurophysiology, and multiple alternative solutions have been proposed during the past few years^1–8^. Some studies on spike sorting have been concerned with simplifying the common steps of sorting processes based on mathematical transformations of the raw neural recording to obtain a new signal that would discriminate among spike waveforms originating from different neurons, which presumably correspond to different groups^9–11^. With that approach, the common spike-detection and spike-identification steps have been simplified, reducing the computational costs in function of their execution times, but other non-common steps (e.g., raw signal segmentation, local maxima selection, and noisy spike discrimination) were inevitably introduced in the spike-sorting process. In other published works, the focus of attention has been on the feature extraction methods^1–3,12–23^. Too often, misapplication of the feature extraction step leads to an extreme reduction of dimensionality and, therefore, the resulting feature matrices correspond with “abstract” mathematical entities (based on coefficients, factors, or components) that do not reflect the main functional properties of the neural events under study.

However, some of the works most cited^7,24–32^ on spike sorting continue to focus on the effort to develop robust and non-redundant spike-sorting algorithms based on the exhaustive extraction of features with a clear physiological description of the spike event. This physiological information of the spike event is highly appreciated in the qualitative and quantitative characterization of the neuronal activity (intracellular, extracellular, or multi-electrode-array recordings) and has practical uses in neurophysiology beyond the mere spike classification^29,33–38^. In particular, extracellular microelectrode recordings can include action potentials from multiple neurons. As the microelectrode tip is surrounded by many neurons, it detects the occurrence of the electrical events generated by all nearby neurons. To separate spikes from different neurons, they can be sorted according to a systematic comparison among spike feature vectors.

In this work, we present an unsupervised spike-sorting method based on shape (features from spike waveform first derivative in time domain), phase (features from spike trajectory in phase space: first derivative vs. second derivative), and distribution features (features from spike amplitude distribution function for both the first and second derivatives) of each spike event, all of them linearly independent and with a proper physiological description. In addition, we compare our spike-sorting method based on shape, phase, and distribution features (SS-SPDF method) with other published methods^1–4,12–22^ also based on feature extraction (see Table 1 for details). By using a dimensionally flexible vector of derivative-based features from each spike event in combination with validity and error indices, spike events can be automatically classified applying the sequence of first, *K*-means^39^ for sorting the single-unit spikes (assigning each of them to its corresponding single-unit cluster) and for identifying the overlapping waveforms, and then, template optimization in the phase space^40–43^ for sorting the subsets of identified overlapping waveforms —that is, *K*-means and template optimization in phase space (*K*-TOPS clustering algorithm).

**Table 1:** Overview of other spike-sorting methods/algorithms based on feature extraction.

Author/Year	Number of Features/Description		Classification Method/Algorithm
Gibson *et al*.^17,18^	2	Areas under the positive (integral I_P_) and negative (integral I_N_) phases of the action potential—that is, the Integral Transform (IT).	Fuzzy C-means clustering.
Jahanmiri-Nezhad *et al*.^4^	2	Peak-to-valley amplitude of the action potential, and the area under the curve (sum of absolute values).	*K*-means clustering + Gaussian mixture model estimation.
Kamboh & Mason^3^; Saeed & Kamboh^21^	2	Zero-Crossing Features (ZCF) of the spike. ZC1 (the sum of all the values before zero-crossing) and ZC2 (the sum of values after zero-crossing).	*K*-means clustering + Mahalanobis distance.
Zviagintsev *et al*.^15^	2	Integral Transform (IT). Discrete and normalized spike integrals I_P_ and I_N_.	Segmented Principal Component algorithm + Principal Component Analysis (PCA).
Lewicki^13^	3	Positive peak amplitude of the spike, peak-to-valley amplitude, and the waveform duration.	*K*-means clustering + Euclidean distance and Scaled Principal Component score.
Yang *et al*.^16^	3	Positive peak amplitude of the spike, and the positive [F_14_] and negative peaks of the spike first derivative (FD).	Spike derivative-based feature extraction algorithm. Spike height + Peaks of spike derivatives.
Paraskevopoulou *et al*.^19,20^	3	Positive peak [F_14_] of the spike FD, and the positive [F_18_] and negative [F_19_] peaks of the spike second derivative (SD).	10-iteration *K*-means clustering + Squared Euclidean metric.
Yang *et al*.^23^	3	Integral of repolarization (IR) of the spike, and the positive [F_14_] and negative peaks of the spike FD.	20-iteration *K*-means clustering + Euclidean metric.
Balasubramanian & Obeid^1^	5	Spike power, spike amplitude range, negative and positive deflections, and the spike gradient slope.	Fuzzy logic-based feature extraction system.
Sonoo & Stalberg^12^	5	Peak-to-valley amplitude of the spike, waveform duration, negative Integral Transform (nIT), ratio (nIT/maximum peak), and the logarithm of the maximum rise of the spike.	Nearest-neighbor Methods + Discriminant Analysis (DA).
Su *et al*.^22^	6	Spike peak amplitude, peak roundness (i.e., the spike peak [F_19_] of the SD), the root-mean-square of pre-spike amplitude, the highest repolarization rate, the afterhyperpolarization (i.e., afterspike minimum), and the correlation coefficient between the spike and the reference waveform.	*K*-means clustering + Principal Component Analysis (PCA).
Bestel^2^	7	Positive and negative peaks of the action potential, left and right spike angles, negative and positive signal energy of a continuous-time signal, and the core spike duration.	Expectation maximization (EM) method + Gaussian basis functions.
Stewart *et al*.^14^	7	Peak-to-valley amplitude of the spike, waveform duration, trailing waveform duration, leading waveform aspect ratio, trailing waveform aspect ratio, waveform transition slope, and event duration.	Nearest-neighbor Methods + Discriminant Analysis (DA).

Note that in general, the authors cited here have used only three (F_14_, F_18_ and F_19_ common features) of the 24 features (F_1_–F_24_) proposed in this work (see Table 3).

The efficiency and reliability of the proposed SS-SPDF feature extraction method and of our *K*-TOPS clustering algorithm (hereinafter SS-SPDF method/algorithm) were demonstrated on both simulated datasets and real extracellular recordings. We have applied here the SS-SPDF method/algorithm by examining their performances in sorting spike events from extracellular recordings at the rostral-medial prefrontal cortex (rmPFC) of rabbits during a paradigm of classical eyeblink conditioning (a special type of associative learning). We clustered the spiking events (single-unit spikes and overlapping waveforms from multi-unit recordings) employing the *K*-TOPS clustering algorithm with three different internal validation indices: Silhouette^44^, Davies-Bouldin^45^, and Dunn^46^. For an objective assessment of the spike-sorting capabilities of the proposed SS-SPDF method/algorithm, two integrative measures of cohesion-dispersion (*CD*-index) and clustering error (*CE*-index) during classification were also implemented. These validity and error indices allowed us to obtain the optimal number of clusters and the optimal clustering, respectively. Therefore, the proposed SS-SPDF method/algorithm seem to be suitable for the off-line analysis of extracellular recordings. Moreover, they could substantially improve the quality of data evaluation based on microelectrode recordings for various applications in neurophysiology.

## Results

### Spike-Sorting Methodology Overview

**Figure 1:**
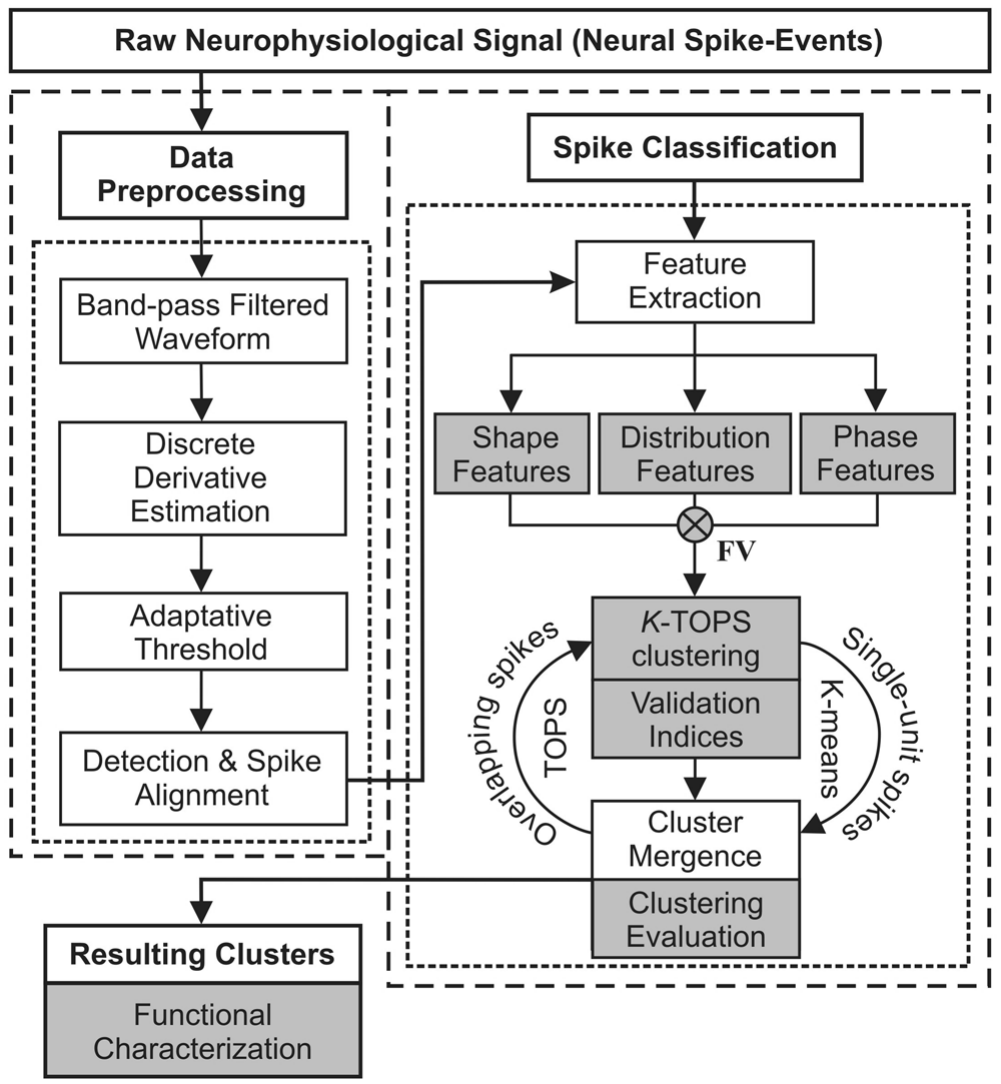
Overall structure of the proposed SS-SPDF method/algorithm. Step-by-step illustration of the spike-sorting process based on shape, phase and distribution features (SS-SPDF method) and *K*-TOPS clustering (*K*-means and template optimization in phase space) with validity and error indices. White sub-blocks represent the common steps of a spike-sorting algorithm. Gray sub-blocks indicate the main methodological contributions of the proposed spike sorting approach, —that were the SS-SPDF method of feature extraction and the *K*-TOPS clustering algorithm for systematically sorting both single-unit spikes and overlapping waveforms. Notice that, shape features refer to measures extracted from spike waveform in time domain of the first derivative, phase features refer to measures extracted from spike trajectory in phase-space (spike first derivative vs. spike second derivative), and distribution features concern to features extracted from spike amplitude distribution function for both the first and second derivatives. At the last step (resulting clusters), a summary subblock (also in gray) for reporting the relevant information of the whole process was implemented. This approach facilitates the physiological interpretation of the extracted spike features, the assessment of the modulating properties of the involved neurons, and the functional characterization of the neural process under study.

The proposed spike-sorting methodology is illustrated graphically in Fig. 1. The block diagram shows the two main steps: (I) data preprocessing (Fig. 1 left block) including the data filtering, the derivative estimation, the amplitude threshold selection, and the spike-detection and alignment steps and (II) spike classification (Fig. 1 right block) including feature extraction and *K*-TOPS clustering steps.

**Figure 2:**
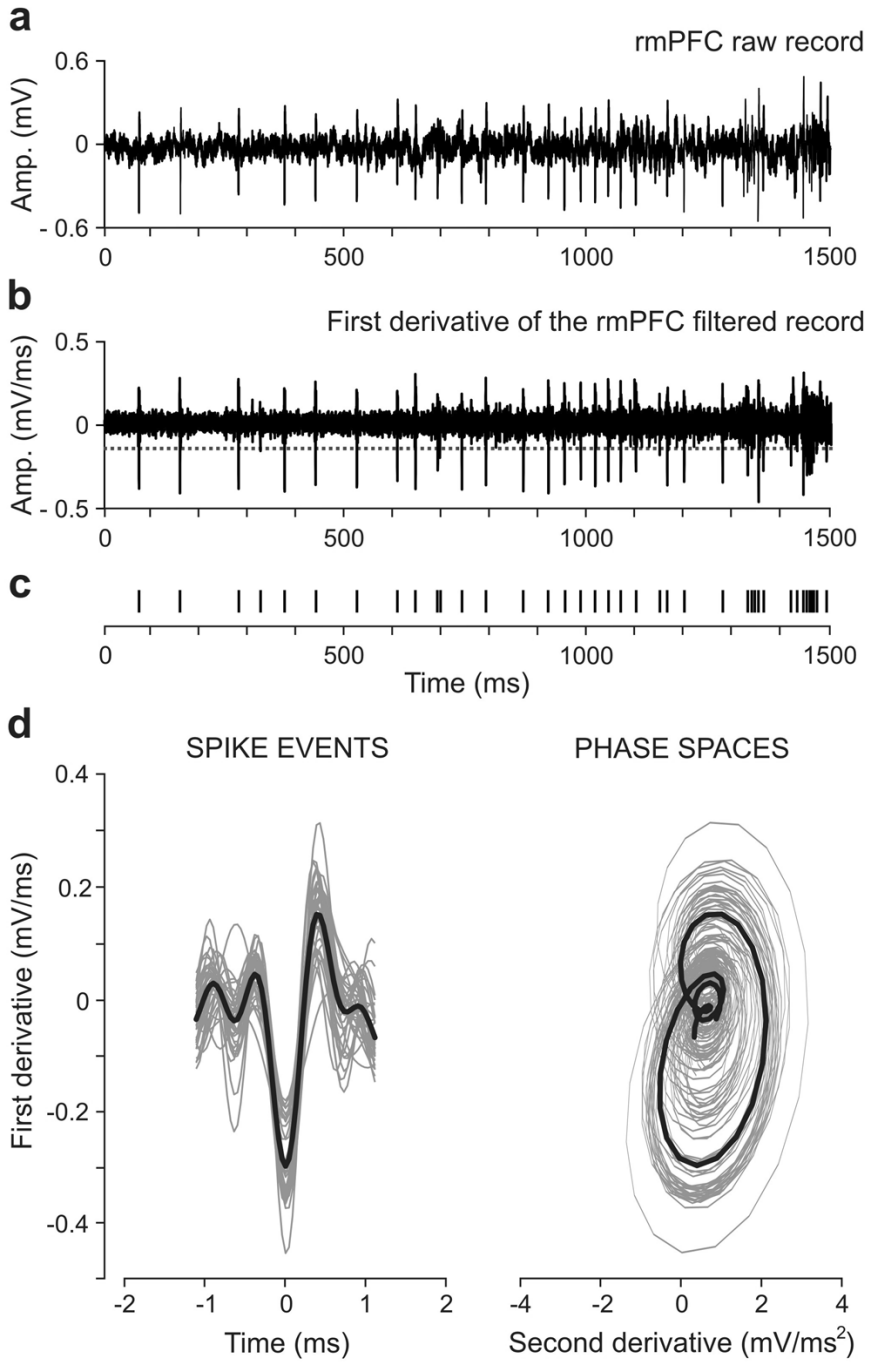
An example illustrating the preprocessing steps during the spike sorting of extracellular rmPFC recordings. (**a**) rmPFC raw record, with 37500 timepoints that corresponds to 1.5 s at a sampling rate of 25 kHz. (**b**) First derivative of the rmPFC band-pass (FIR filter, 450–2050 Hz) filtered record. The horizontal dotted line indicates an alternative amplitude threshold for direct spike-event detection. (**c**) Distribution in time of the spikes detected in **b**. (**d**) Left, resulting spike events (gray traces) in the time domain (time vs. first derivative) and their corresponding mean event profile (black trace). Right, phase space portraits (second derivative vs. first derivative; gray traces) of the spikes and their corresponding mean phase trajectory (black trace).

During the preprocessing (see Signal Preprocessing Overview section in Methods), we adopted the same scheme that other authors^19,20,40–42^ whose have applied a derivative-based criterion [see Fig. 2 and Eq. (1) in Methods] for determining the adaptive threshold. According to this, the prior calculation of the first derivative of the recording for the subsequent selection of the amplitude threshold did not affect the data preprocessing and therefore the spike detection step retained the same completeness as for other standard methods —i.e., the optimal performance of both steps (amplitude threshold selection and spike detection, Fig. 2) was guaranteed in this study.

**Figure 3:**
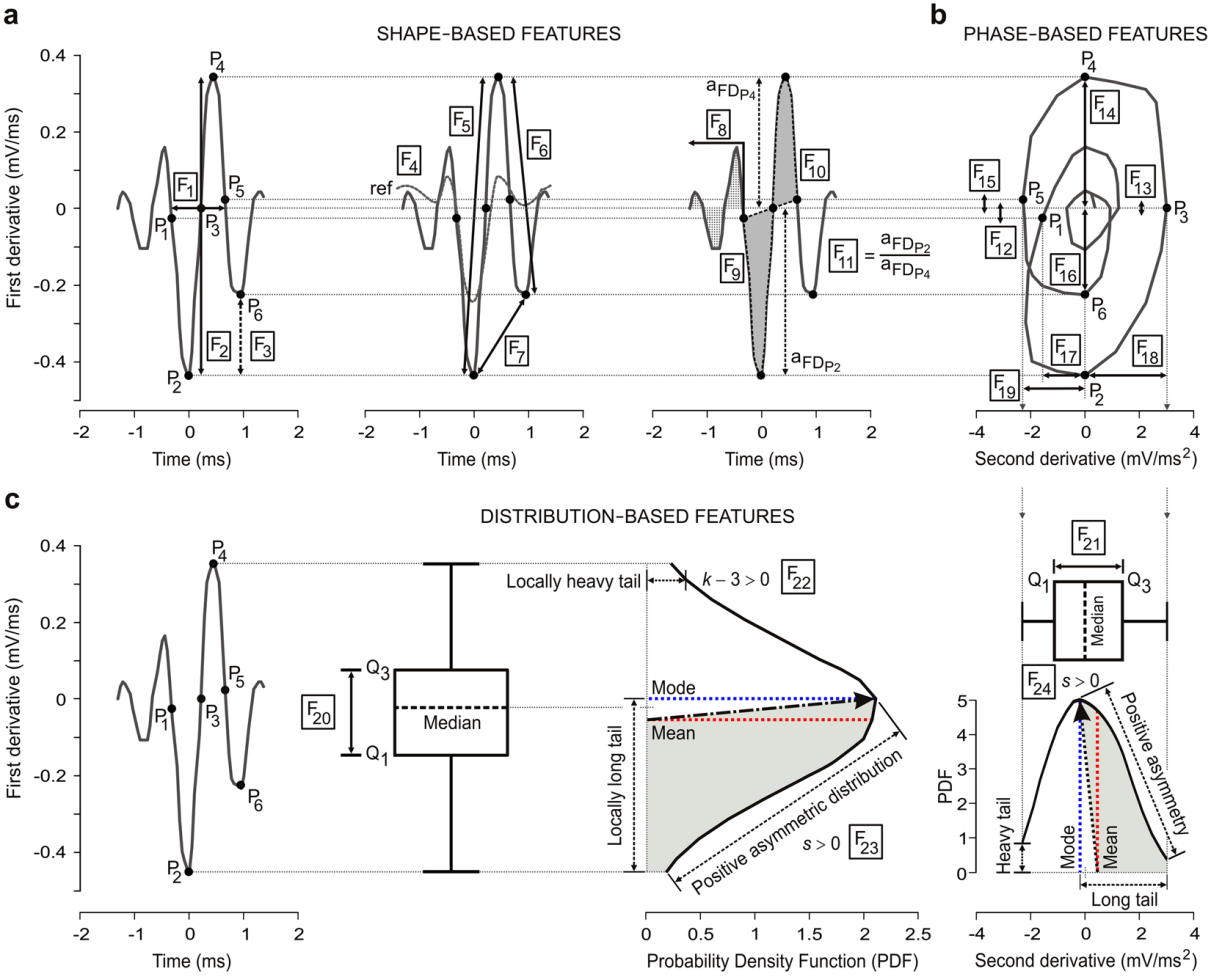
Schematic representation of the feature-extraction method. (**a**) Six fundamental points (P_1_–P_6_, see Table 2) and 11 shape-based features (F_1_–F_11_) from each spike event in the time domain of the spike first derivative (FD). (**b**) Eight phase-based features (F_12_–F_19_) from each spike trajectory in the phase space (second derivative (SD) vs. FD). (**c**) Five distribution-based features (F_20_–F_24_) for the statistical amplitude distribution of the FD (i.e., F_20_, F_22_, and F_23_) and SD (i.e., F_21_ and F_24_) of the spike. Note that for each spike amplitude distribution (probability density function, PDF), the mean, median, mode, interquartile range (Q_3_–Q_1_), kurtosis (e.g., *k* – 3 > 0), and asymmetry (e.g., *s* > 0) are indicated. In summary, a vector of 24 features (F_1_–F_24_, see Table 3) was determined for each spike event.

During the feature extraction (see Feature Extraction section in Methods), we extracted a vector of twenty-four physiological features (Fig. 3, Tables 2 and 3) for each spike event. According to the proposed SS-SPDF method of feature extraction, each 24D-vector included shape (features from spike waveform first derivative in time domain), phase (features from spike trajectory in phase space: first derivative vs. second derivative), and distribution (features from spike amplitude distribution function for both the first and second derivatives) features. All these vectors of independent derivative-based features (i.e., independent because there is not multi-collinearity among them, see algebraic definitions in Table 3) were dimensionally flexible —i.e., each feature subset of this vector [which was selected according to the criterion of the Eq. (2), see Methods] was an appropriate independent-feature vector. The resulting matrix of feature vectors constitutes the input to the clustering procedure that performed the classification of the spike events in different clusters and assigned each cluster to a neuronal unit.

**Table 2:** List of the selected waveform components.

Spike-points number	Definition
P_1_	First zero-crossing of the FD before the action potential has been detected
P_2_	Valley of the FD of the action potential
P_3_	Second zero-crossing of the FD of the action potential that has been detected
P_4_	Peak of the FD of the action potential
P_5_	Third zero-crossing of the FD after the action potential has been detected
P_6_	Valley of the FD after the action potential

Six fundamental points (P_1_–P_6_) determined each detected spike. These points were graphically identified in both time domain (Fig. 3a) and phase space (see Fig. 3b), considering the first derivative (FD) and the second derivative (SD) of the action potential.

**Table 3:** Neurophysiological features of each spike characterizing the process of creating objects (24D feature-vectors).

	Number	Name	Algebraic definition
Shape	F_**1**_	Waveform duration of the FD of the action potential	t_P5_ − t_P1_
	F_**2**_	Peak-to-valley amplitude of the FD of the action potential	${{\rm{a}}}_{{{\rm{FD}}}_{{\rm{P}}4}}-{{\rm{a}}}_{{{\rm{FD}}}_{{\rm{P}}2}}$
	F_**3**_	Valley-to-valley amplitude of the FD of the action potential	${{\rm{a}}}_{{{\rm{F}}{\rm{D}}}_{{\rm{P}}6}}-{{\rm{a}}}_{{{\rm{F}}{\rm{D}}}_{{\rm{P}}2}}$
	F_**4**_	Correlation coefficient between the FD of the action potential (ap) and the reference spike-waveform (ref), considering their corresponding standard deviation *σ*_FD_	$\frac{{\sigma }_{{{\rm{FD}}}_{\mathrm{ap},\mathrm{ref}}}^{2}}{{\sigma }_{{{\rm{FD}}}_{{\rm{ap}}}}\cdot {\sigma }_{{{\rm{FD}}}_{{\rm{ref}}}}}\,\,$
	F_**5**_	Logarithm of the positive deflection of the FD of the action potential	$\mathrm{log}(\frac{{{\rm{a}}}_{{{\rm{FD}}}_{{\rm{P}}4}}-{{\rm{a}}}_{{{\rm{FD}}}_{{\rm{P}}2}}}{{{\rm{t}}}_{{\rm{P}}4}-{{\rm{t}}}_{{\rm{P}}2}})$
	F_**6**_	Negative deflection of the FD of the action potential	$\frac{{{\rm{a}}}_{{{\rm{FD}}}_{{\rm{P}}6}}-{{\rm{a}}}_{{{\rm{FD}}}_{{\rm{P}}4}}}{{{\rm{t}}}_{{\rm{P}}6}-{{\rm{t}}}_{{\rm{P}}4}}$
	F_**7**_	Logarithm of the slope among valleys of the FD of the action potential	$\mathrm{log}(\frac{{{\rm{a}}}_{{{\rm{FD}}}_{{\rm{P}}6}}-{{\rm{a}}}_{{{\rm{FD}}}_{{\rm{P}}2}}}{{{\rm{t}}}_{{\rm{P}}6}-{{\rm{t}}}_{{\rm{P}}2}})$
	F_**8**_	Root-mean-square of pre-event amplitudes of the FD of the action potential	$\sqrt{\frac{{{\rm{a}}}_{{{\rm{FD}}}_{{\rm{P}}1}}+{\sum }_{i\,=\,m-1}^{1}\,{{\rm{a}}}_{i}}{m}}$
	F_**9**_	Negative slope ratio of the FD of the action potential	$(\frac{{{\rm{a}}}_{{{\rm{FD}}}_{{\rm{P}}2}}-{{\rm{a}}}_{{{\rm{FD}}}_{{\rm{P}}1}}}{{{\rm{t}}}_{{\rm{P}}2}-{{\rm{t}}}_{{\rm{P}}1}})/(\frac{{{\rm{a}}}_{{{\rm{FD}}}_{{\rm{P}}3}}-{{\rm{a}}}_{{{\rm{FD}}}_{{\rm{P}}2}}}{{{\rm{t}}}_{{\rm{P}}3}-{{\rm{t}}}_{{\rm{P}}2}})$
	F_**10**_	Positive slope ratio of the FD of the action potential	$(\frac{{{\rm{a}}}_{{{\rm{FD}}}_{{\rm{P}}4}}-{{\rm{a}}}_{{{\rm{FD}}}_{{\rm{P}}3}}}{{{\rm{t}}}_{{\rm{P}}4}-{{\rm{t}}}_{{\rm{P}}3}})/(\frac{{{\rm{a}}}_{{{\rm{FD}}}_{{\rm{P}}5}}-{{\rm{a}}}_{{{\rm{FD}}}_{{\rm{P}}4}}}{{{\rm{t}}}_{{\rm{P}}5}-{{\rm{t}}}_{{\rm{P}}4}})$
	F_**11**_	Peak-to-valley ratio of the FD of the action potential	$\frac{{{\rm{a}}}_{{{\rm{FD}}}_{{\rm{P}}2}}}{{{\rm{a}}}_{{{\rm{FD}}}_{{\rm{P}}4}}}$
Phase	**F**_**12**_	Amplitude of the FD of the action potential relating to P_1_	${{\rm{a}}}_{{{\rm{FD}}}_{{\rm{P}}1}}$
	F_**13**_	Amplitude of the FD of the action potential relating to P_3_	${{\rm{a}}}_{{{\rm{FD}}}_{{\rm{P}}3}}$
	F_14_*	Amplitude of the FD of the action potential relating to P_4_	${{\rm{a}}}_{{{\rm{FD}}}_{{\rm{P}}4}}$
	**F**_**15**_	Amplitude of the FD of the action potential relating to P_5_	${{\rm{a}}}_{{{\rm{FD}}}_{{\rm{P}}5}}$
	F_**16**_	Amplitude of the FD of the action potential relating to P_6_	${{\rm{a}}}_{{{\rm{FD}}}_{{\rm{P}}6}}$
	F_**17**_	Amplitude of the SD of the action potential relating to P_1_	${{\rm{a}}}_{{{\rm{SD}}}_{{\rm{P}}1}}$
	**F**_**18**_ *	Amplitude of the SD of the action potential relating to P_3_	${{\rm{a}}}_{{{\rm{SD}}}_{{\rm{P}}3}}$
	F_19_*	Amplitude of the SD of the action potential relative to P_5_	${{\rm{a}}}_{{{\rm{SD}}}_{{\rm{P}}5}}$
Distribution	F_**20**_	Inter-quartile range (Q_3_ − Q_1_) of the FD of the action potential, considering the percentiles ${{\rm{P}}}_{{75}_{{\rm{FD}}}}$ and ${{\rm{P}}}_{{25}_{{\rm{FD}}}}$	${{\rm{P}}}_{{75}_{{\rm{FD}}}}-{{\rm{P}}}_{{25}_{{\rm{FD}}}}$
	F_**21**_	Inter-quartile range (Q_3_ − Q_1_) of the SD of the action potential, considering the percentiles ${{\rm{P}}}_{{75}_{{\rm{SD}}}}$ and ${{\rm{P}}}_{{25}_{{\rm{SD}}}}$	${{\rm{P}}}_{{75}_{{\rm{SD}}}}-{P}_{{25}_{{\rm{SD}}}}$
	F_**22**_	Kurtosis coefficient of the FD of the action potential, considering the fourth sampling moment of *n* amplitudes ${{\rm{a}}}_{{{\rm{FD}}}_{i}}$ about its mean $\overline{{{\rm{a}}}_{{\rm{FD}}}}$, and the standard deviation *σ*_FD_	$\,\frac{{\sum }_{i=1}^{n}\,{({{\rm{a}}}_{{{\rm{FD}}}_{i}}-\overline{{{\rm{a}}}_{{\rm{FD}}}})}^{4}}{n\cdot {\sigma }_{{\rm{FD}}}^{4}}$
	F_**23**_	Fisher asymmetry of the FD of the action potential, considering the third sampling moment of *n* amplitudes ${{\rm{a}}}_{{{\rm{FD}}}_{i}}$ about its mean $\overline{{{\rm{a}}}_{{\rm{FD}}}}$, and the standard deviation *σ*_FD_	$\frac{{\sum }_{i=1}^{n}\,{({{\rm{a}}}_{{{\rm{FD}}}_{i}}-\overline{{{\rm{a}}}_{{\rm{FD}}}})}^{3}}{n\cdot {\sigma }_{{\rm{FD}}}^{3}}$
	F_**24**_	Fisher asymmetry of the SD of the action potential, considering the third sampling moment of *n* amplitudes ${{\rm{a}}}_{{{\rm{FD}}}_{i}}$ about its mean $\overline{{{\rm{a}}}_{{\rm{FD}}}}$, and the standard deviation *σ*_SD_	$\frac{{\sum }_{i=1}^{n}\,{({{\rm{a}}}_{{{\rm{SD}}}_{i}}-\overline{{{\rm{a}}}_{{\rm{SD}}}})}^{3}}{n\cdot {\sigma }_{{\rm{SD}}}^{3}}$

List of shape (F_1_–F_11_), phase (F_12_–F_19_), and distribution (F_20_–F_24_) features and their algebraic definition (see graphic representation in Fig. 3), considering the first derivative (FD) and the second derivative (SD) of each action potential. The three common features (F_14_, F_18_ and F_19_) proposed also by other authors^19,20^ (see Table 1) are marked with an asterisk.

During the spike event clustering (see Clustering Algorithm section in Methods), the number of spikes distributed across time and their neuronal identity was determined applying the sequence of first, an unsupervised *K*-means clustering for sorting the single-unit spikes, and then an algorithm of template optimization in phase space for sorting the overlapping waveforms (that is, the proposed *K*-TOPS clustering algorithm). In this classification process we use three internal validation measures (Silhouette^44^, Davies-Bouldin^45^ and Dunn^46^ indices) and two customized validity [*CD*-index, Eqs (3–6)] and error [*CE*-index, Eqs (7–9)] indices, which are described in detail in Methods and in Supplementary Material.

In this way, spike events (single-unit spikes and overlapping waveforms) originated from different neurons—which presumably correspond to different clusters—could be classified and properly sorted. Moreover, the cohesion-dispersion among and within clusters during the neural events classification could also be studied. Validity and error indices were tested for the proposed SS-SPDF method/algorithm in comparison with other feature extraction methods, and the results were compared appropriately, taking into account the quality of the spike sorting on both simulated datasets (see Simulated Data section in Methods) and real extracellular recordings (see Experimental Data section in Methods).

### Application and Validation of SS-SPDF Method/Algorithm on Simulated Data

In order to verify and validate the proposed spike-sorting method/algorithm, simulated data (see Methods), without noise and with added noise^40–42^, were analyzed. Simulated data (sampling frequency of 44 kHz and duration of 180 s) were very similar to those reported by other authors^24^ [with three spike templates (*T*1, *T*2 and *T*3) and an important noise component] but in their study only 100 spikes from each neuron at an average rate of 30 spikes/s were analyzed, containing 19 superpositions. In this study, we added 2700 instances of each template randomly to the background noise, avoiding template overlapping. The resulting spike train mimicked three neurons firing independently at an average rate of 15 spikes/s and contained 81 overlapping waveforms. The rationale for using a larger simulated dataset is to provide a more robust test of the proposed SS-SPDF method/algorithm. Validation tests on simulated data that closely resemble real recordings allow the systematic study of tolerance to noise and evaluates whether the algorithm can detect the exact number of clusters and what is the misclassification rate among clusters.

**Figure 4:**
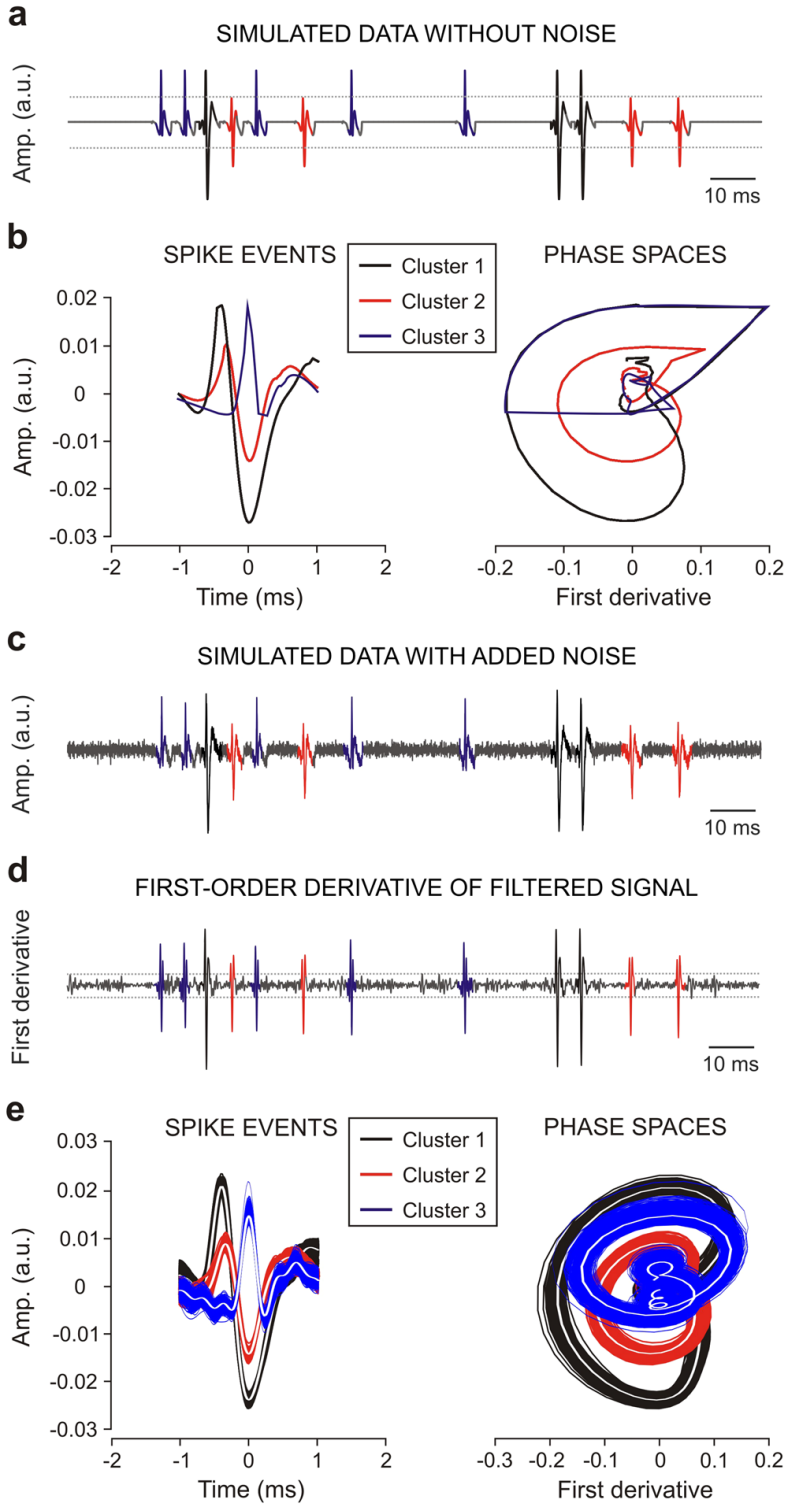
Validation on simulated data. The wide-ranging simulated data were sampled at 44 kHz during 180 s. The time-window of the represented simulated data (panels a and c) was of 150 ms. (**a**) Simulated data without noise. (**b**) At the left, it is represented the three activity patterns (cluster 1, black template; cluster 2, red template; and cluster 3, blue template) detected from the wide-ranging simulated data with action potentials clustering by SS-SPDF method/algorithm. At the right, it is represented their corresponding phase-space portraits (action potential vs. its first derivative). (**c**) Simulated data with low added noise and a signal-to-noise ratio of 3.55 dB. (**d**) First derivative of the band-pass (FIR filter, 450–2600 Hz) filtered signal. (**e**) At the left, it is represented the three activity patterns (cluster 1, 2728 spikes, black traces; cluster 2, 2690 spikes, red traces; and cluster 3, 2733 spikes, blue traces) of the wide-ranging (180 s) simulated data with action potentials sorting by SS-SPDF method/algorithm (comprising *K*-TOPS clustering), while the phase-space portraits of them are represented at the right part of this panel. Here, each white trace (resulting template) represents the mean spike-event of each cluster.

The first step was to test our SS-SPDF method/algorithm on a simulated data without noise (Fig. 4a). The simulated action potentials were directly detected and aligned without preprocessing because the algorithm was implemented in a no-noise situation. In particular, the detection of the simulated action potentials was performed applying two amplitude thresholds (horizontal dotted lines in Fig. 4a) which were manually selected. For each simulated action potential, the 24D-vector of features (see Table 3) was determined and the classification process for all the spike events was executed. The results allowed to show three activity patterns (cluster 1, cluster 2 and cluster 3, in Fig. 4b) for the simulated data. In addition, the phase space portraits (PSPs; action potential vs. its first derivative) are represented in the right part of Fig. 4b.

For the second validation step, the SS-SPDF method/algorithm was applied on the same simulated data (44 kHz and 180 s) but with low level of added noise (signal-to-noise ratio of 3.55 dB)^41^. To perform the preprocessing in this situation with added noise (Fig. 4c), the simulated data was filtered with a bandpass FIR filter from 450 to 2600 Hz (spike durations ranging from 0.4 to 2.2 ms). In addition, the first-order derivative of the filtered record (Fig. 4d) was calculated and the spike events were detected and aligned. Two amplitude thresholds (horizontal dotted lines in Fig. 4d) were automatically selected according to Eq. (1) (see Methods). In the same way, for each simulated action potential, the 24D-vector of features was determined and the classification process for all the spike events was implemented. In this situation (simulated data with added noise), the results also allowed to show three activity patterns (cluster 1, 2728 spikes; cluster 2, 2690 spikes; and cluster 3, 2733 spikes in Fig. 4e). The three activity patterns of the simulated action potentials are represented at the left part of Fig. 4e, while their phase space portraits (PSPs; action potential versus its first derivative) are represented at the right part of the Fig. 4e. White traces (spike templates) are indicating the mean spike events for the identified clusters.

The results obtained after performing the two validation tests on simulated data (with and without noise) were similar —i.e., three clusters of activity patterns. The three identified clusters allowed to determine the three templates of spike events (color traces in Fig. 4b and white traces in Fig. 4e), which were in perfect correspondence to those three default templates (*T*1, *T*2 and *T*3) of the simulated data with embedded simulated spikes obtained by other authors^24,40,41^. In summary, Table 4 shows the observed classification matrices resulting from our SS-SPDF method/algorithm [comprising *K*-TOPS clustering, —that is, applying the sequence of first, *K*-means (for sorting the single-unit spikes), and then, template optimization in the phase space (for sorting the overlapping waveforms)]. In addition, the numbers and percentages (%) of well-classified, misclassified and unclassified spikes events and the Error Index values were also informed. These Error Index values were calculated according to Eq. (9) for five simulated datasets (from D_1 to D_5), which were obtained by the overlap of the different templates with time shift sequences smaller than 2 ms. The number of well-classified spikes ranged 2725–2731 for cluster 1 (Mean ± SEM; 2728 ± 1.1 events), 2685–2695 for cluster 2 (2690 ± 1.8 events) and 2730–2735 for cluster 3 (2733 ± 1.0 events), according to the diagonal values of the observed classification matrices (see Table 4). SS-SPDF method/algorithm were able to separate types *T*1, *T*2 and *T*3 into distinct clusters. Cluster 1, 2 and 3 proved highly specific for types *T*1, *T*2 and *T*3, respectively, because the number of misclassified spikes was small (*K*-TOPS, 13.0 ± 1.1 events) ranging between 11 and 17 events. The number of unclassified spike events (*K*-TOPS, 17.0 ± 2.9 events) ranging between 9 and 18 events was comparable to the number of misclassified spike events for our simulated datasets with a duration of 81 s and 8181 spike events (8100 single-unit spikes and 81 overlapping waveforms). This amount of unclassified profiles (*K*-TOPS, 17.0 ± 2.9 events) was clearly lower than those reported by other authors^24^ [Wavelet-based Spike Classifier (WSC), 33.4 ± 8.6 events, ranging between 21 and 64 events], even when they employed a simulated spike train with a shorter time window (3.2 s) and fewer spike events (300 instances, 19 superpositions) —that is, in percentage terms, an 11% with WST method and only a 0.2% of unclassified profiles with our SS-SPDF method/algorithm (including *K*-TOPS clustering approach). Notice that, our *K*-TOPS clustering algorithm correctly classified the 99.63% of the spike events (8151 of a total of 8181), the 99.80% of the single-unit spikes (8084 of a total of 8100) and the 87.72% of the overlapping waveforms (67 of a total of 81), which means an error index of *EI* = 22.634 ± 1.665 (Mean ± SEM; see Table 4).

**Table 4:** Observed classification matrices resulting from our SS-SPDF method/algorithm [*K*-TOPS approach —that is, applying the sequence of first, *K*-means (for sorting the single-unit spikes), and then, template optimization in the phase space (for sorting the overlapping waveforms)] on five simulated datasets (from D_1 to D_5).

Dataset	Classification Matrix	*Unclassified	*Misclassified	*Well-classified	Error Index
D_1	$$\begin{array}{c}\,\,T1\,\,T2\,\,T3\\ \begin{array}{c}{C}_{1}\\ {C}_{2}\\ {C}_{3}\end{array}(\begin{array}{ccc}2730 & 5 & 1\\ 6 & 2685 & 1\\ 0 & 0 & 2730\end{array})\end{array}$$	23 (13)	13 (4)	8145 (64)	26.665
D_2	$$\begin{array}{c}\,\,T1\,\,T2\,\,T3\\ \begin{array}{c}{C}_{1}\\ {C}_{2}\\ {C}_{3}\end{array}(\begin{array}{ccc}2728 & 4 & 1\\ 5 & 2695 & 1\\ 0 & 0 & 2735\end{array})\end{array}$$	12 (9)	11 (1)	8158 (71)	17.436
D_3	$$\begin{array}{c}\,\,T1\,\,T2\,\,T3\\ \begin{array}{c}{C}_{1}\\ {C}_{2}\\ {C}_{3}\end{array}(\begin{array}{ccc}2731 & 6 & 1\\ 5 & 2691 & 1\\ 0 & 0 & 2733\end{array})\end{array}$$	13 (9)	13 (2)	8155 (70)	20.518
D_4	$$\begin{array}{c}\,\,T1\,\,T2\,\,T3\\ \begin{array}{c}{C}_{1}\\ {C}_{2}\\ {C}_{3}\end{array}(\begin{array}{ccc}2726 & 5 & 1\\ 4 & 2687 & 1\\ 0 & 0 & 2732\end{array})\end{array}$$	25 (18)	11 (1)	8145 (62)	25.357
D_5	$$\begin{array}{c}\,\,T1\,\,T2\,\,T3\\ \begin{array}{c}{C}_{1}\\ {C}_{2}\\ {C}_{3}\end{array}(\begin{array}{ccc}2725 & 10 & 1\\ 5 & 2692 & 1\\ 0 & 0 & 2735\end{array})\end{array}$$	12 (11)	17 (2)	8152 (68)	23.195
Mean	$$\begin{array}{c}\,\,T1\,\,T2\,\,T3\\ \begin{array}{c}{C}_{1}\\ {C}_{2}\\ {C}_{3}\end{array}(\begin{array}{ccc}2728 & 6 & 1\\ 5 & 2690 & 1\\ 0 & 0 & 2733\end{array})\end{array}$$	17 (12) Percentage (%) 0.21% (14.81%)	13 (2) Percentage (%) 0.16% (2.47%)	8151 (67) Percentage (%) 99.63% (82.72%)	22.634 SEM 3.724

The first number in unclassified, misclassified or well-classified columns is the number of spike events. The number in parentheses represents the number of overlapping waveforms for each category. In the last row, the corresponding percentage values (%) are indicated. In the last column, the resulting Error Index [see Eq. (9)] for each simulated dataset and the average value of them are reported. ^*^Here are indicated, well-classified (${w}_{S}=\sum {d}_{i}$), misclassified (${m}_{S}=\sum {r}_{k}$) and unclassified [${u}_{S}={N}_{S}-(\sum {d}_{i}+\sum {r}_{k}$)] spike events. Also, *N*_*S*_ is the total number of spike events (8181, among which 8100 are single-unit spikes and 81 are overlapping waveforms), while *d*_*i*_ and *r*_*k*_ are the diagonal and nondiagonal elements of the observed classification matrix, respectively. Abbreviations: SEM, standard error of the mean.

On the other hand, to assess the sorting capabilities of the proposed *K*-TOPS clustering algorithm on simulated data and to compare it with other methods [Principal Component Analysis (PCA), Reduced Feature Set (RFS), Wavelet-based Spike Classifier (WSC), and Template-Matching in Phase Space (TMPS)], we reported in Table 5 the Error Index values, which were also calculated according to Eq. (9). The Error Index ranks our *K*-TOPS clustering as best performer on simulated spike train, followed by the WSC, RFS and PCA. The PCA method showed the worst performance because its algorithm failed to correctly distinguish three classes and returned the highest number of misclassified events (PCA, 94 ± 1.3 events) ranging between 92 and 99 events. In general, both WSC and SS-SPDF methods clearly separated the complete population into three classes that are quite specific for the three types *T*1, *T*2 and *T*3. However, during the spike classification with WSC method, the set of unclassified events contained mostly the short-delay superpositions. This was one of the main reasons that determined an Error Index value (WSC, 35.9 ± 3.2, ranged 30.5–47.1) higher than that obtained with our SS-SPDF method/algorithm (*K*-TOPS, 22.6 ± 1.7, ranged 17.4–26.7). These two Error Index values showed statistically significant differences between them [One-way ANOVA *F*-test between two groups; *F*_(1,8)_ = 13.766; *P* = 0.006].

**Table 5:** Error Index for different approaches: Principal Component Analysis (PCA), Reduce Feature Set (RFS), Wavelet-based Spike Classifier (WSC), *K*-means (*first step alone*), *K*-means and Template Optimization in Phase Space (*K*-TOPS, *two-steps workflow*) and Template-Matching in Phase Space (TMPS), all of them applied to simulated datasets with the same template generation protocol (*T*1, *T*2 and *T*3 in Fig. 4).

Method/Algorithm	Category		Error Index
*Simulated data description (overlapping waveforms were generated as superpositions of the single-unit templates *T*1, *T*2 and *T*3, see Fig. 4).	Unclassified	Misclassified	Mean ± SEM
**Principal Component Analysis (PCA)**			
*The resulting spike train mimicked three neurons (with 100 instances per unit) firing independently at an average rate of 30 spikes/s and contained 19 overlapping waveforms.	22.4 ± 4.082 (ranged 8–26)	94.0 ± 1.304 (ranged 92–99)	137.650 ± 0.951 (ranged 135.6–140.8)
**Reduced Feature Set (RFS)**			
*The resulting spike train mimicked three neurons (with 100 instances per unit) firing independently at an average rate of 30 spikes/s and contained 19 overlapping waveforms.	21.4 ± 5.335 (ranged 9–38)	59.8 ± 1.241 (ranged 56–63)	88.620 ± 1.225 (ranged 85.7–92.8)
**Wavelet-based Spike Classifier (WSC)**			
*The resulting spike train mimicked three neurons (with 100 instances per unit) firing independently at an average rate of 30 spikes/s and contained 19 overlapping waveforms.	33.4 ± 8.606 (ranged 20–64)	20.6 ± 1.833 (ranged 14–25)	35.920 ± 3.170 (ranged 30.5–47.1)
***K*** **-means (** ***first step alone*** **) on derivative-based feature vectors**			
*The resulting spike train mimicked three neurons (with 2700 instances per unit) firing independently at an average rate of 15 spikes/s and contained 81 overlapping waveforms.	86.0 ± 1.581 (ranged 82–91)	11.0 ± 1.049 (ranged 9–15)	58.529 ± 0.431 (ranged 57.4–59.7)
***K*-means and Template Optimization in Phase Space (*K*-TOPS**, ***two-steps workflow*)**			
*The resulting spike train mimicked three neurons (with 2700 instances per unit) firing independently at an average rate of 15 spikes/s and contained 81 overlapping waveforms.	17.0 ± 2.881 (ranged 12–25)	13.0 ± 1.095 (ranged 11–17)	22.634 ± 1.665 (ranged 17.4–26.7)
***K*-means and Template Optimization in Phase Space (*K*-TOPS**, ***two-steps workflow*)**			
*The resulting spike train mimicked three neurons (with 2700 instances per unit) firing independently at an average rate of 15 spikes/s and contained 72 overlapping waveforms.	17.6 ± 3.558 (ranged 7–26)	3.4 ± 1.030 (ranged 1–7)	13.848 ± 1.468 (ranged 10.3–17.3)
**Template-Matching in Phase Space (TMPS)**			
*The resulting spike train mimicked three neurons (with 10^4^ instances per unit) firing independently at an average rate of 10 spikes/s, but without considering overlapping waveforms.	Unreported	Unreported	13.010 ± 1.789

The number of both unclassified and misclassified spike events and the Error Index values are reported by the Mean ± SEM (standard error of the mean).

In addition, we showed in Table 5 the advantages of the proposed two-steps workflow (*K*-TOPS algorithm) for sorting both the single-unit spikes (with *K*-means, first step) and the overlapping waveforms (with TOPS, second step). Notice that, the first-step alone yielded an Error Index value (*K*-means, 58.5 ± 0.4, ranged 57.4–59.7) that was also much higher than that obtained with our integrated *K*-TOPS clustering algorithm (22.6 ± 1.7, ranged 17.4–26.7) — i.e., there were statistically significant differences between them [Kruskal-Wallis One-Way ANOVA on Ranks; *H* = 6.818 with 1 degrees of freedom; *P* = 0.008]. It is important to note that, during the spike classification with *K*-means (first step alone, see Supplementary Table S1), the set of unclassified events contained mostly the simulated overlapping waveforms. After comparing the performance in both cases, we could verify that the addition of TOPS step provided a substantial improvement over the first step alone (*K-*means), all of this because with the combination of *K*-means and TOPS steps (*K*-TOPS algorithm) it was possible to properly sort most of the detected spike events (mainly single-unit spikes and overlapping waveforms) and significantly reduce the Error Index from 58.5 (*K-*means alone) to 22.6 (*K*-TOPS). In summary, *K*-TOPS algorithm as measured by the Error Index [see Eq. (9)] outperformed the WSC, Unsupervised *K*-means (first step alone), RFS and PCA algorithms (see Table 5). Simulated overlapping waveforms obtained by combinations of two and three templates with short-delay superpositions that could not be separated or were largely misclassified by these methods, were better classified by the SS-SPDF method/algorithm (comprising the proposed *K*-TOPS clustering algorithm).

Finally, in the last row of Table 5, we have included the comparison with TMPS method^40–42^. The TMPS method applied an unsupervised learning algorithm that estimates the number of classes and their centers according to the distance between spike trajectories in phase space —that is, a template-matching algorithm in phase space, but without any prior sorting (e.g., *K*-means with derivative-based features) of the spike waveform. The disadvantage of the results obtained with TMPS method^40–42^ was that it did not consider the problem raised by spike overlapping —i.e., the overlapping waveforms were discarded during the template-matching in phase space. Interestingly, the Error Index obtained with the TMPS method^40^ (13.0 ± 1.8) was very close to that obtained with the SS-SPDF method/algorithm (*K*-TOPS, 13.8 ± 1.5, ranged 10.3–17.3) when we did not include the simulated overlapping waveforms resulting from spike combination of the templates *T*1 and *T*2, which introduce most of the misclassified spike events (see observed classification matrices in Table 4).

### Application and Validation of SS-SPDF Method/Algorithm on Real Recordings

In this section, the performance of the proposed spike-sorting method on experimental data (see Methods) is evaluated. The SS-SPDF method was tested on extracellular real recordings of the rmPFC of rabbits (n = 3). Spike detection success rates for a single electrode reached 39.51 ± 2.77 spikes from each trial of recording at the rmPFC in an epoch of 1.5 s of duration that included the conditioned stimulus (CS) – unconditioned stimulus (US) interval. The amount of spikes found in that short epoch (≈40 spike, for each CS–US trial) of the electrophysiological recording was sufficient to explain the modulating properties of the involved rmPFC neurons and also to describe qualitatively and quantitatively the neuronal correlates of the learning process (eyeblink classical conditioning) under study^37^. The conditioning session consisted of 60 CS–US trials separated at random by intervals of 60 s. Each session of recording at the rmPFC lasted ≈ 1 h. The spike sorting analysis was performed exclusively on the 60 CS–US trials of each session of rmPFC recordings. The total number of spikes found from all the trials of a conditioning session (≈ 2400 spikes, for 60 epochs of 1.5 s) seems to be appropriate to study sample real populations —i.e., neural data that are in the form of multiple repetitions of relatively short trials. In this study, each trial was considered to be an independent epoch of the extracellular recording during spike classification process. Rabbits were trained on 10 successive conditioning sessions (from C1 to C10). For each detected spike twenty-four physiological features were extracted, based on shape, phase and distribution measures (see Table 3) from the first and second derivatives of the action potential (see Fig. 3). Combining the proposed SS-SPDF method/algorithm (i.e., twenty-four features from spike derivatives in an optimal feature setting with linear independence among them) with other spike feature extraction algorithms, improved spike sorting performance was achieved without using complex training algorithms such as those employed intensively in other spike feature extraction methods^13,47,48^ to properly classify different spike classes that come from a single neuronal unit or to differentiate similar spikes coming from different neurons.

**Figure 5:**
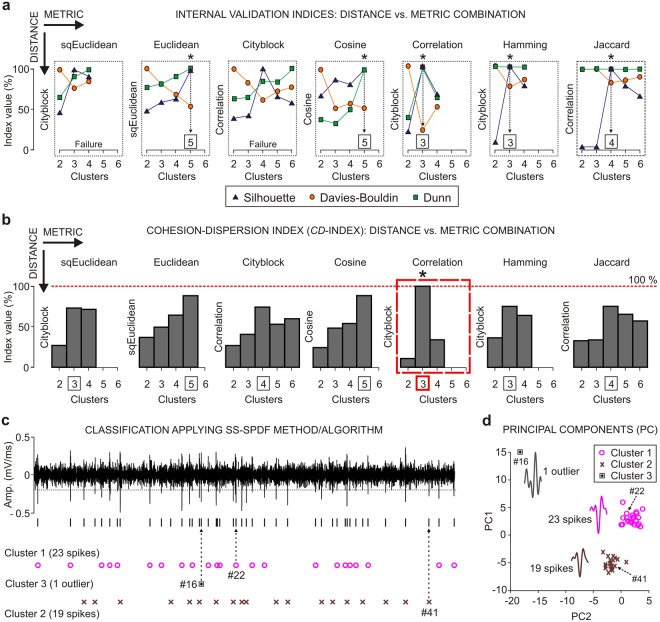
Automatic *K*-means clustering and the cohesion-dispersion index (*CD*-index). (**a**) Some examples (one for each metric) considering different combinations (distance vs metric) for their comparative analysis using the proposed 24D feature vectors (Table 3). Each gray dotted square indicates the relationship between the value (in %) of the internal validation index (Silhouette, blue triangle; Davies-Bouldin, orange circle; or Dunn, green square) and the number of clusters obtained by the unsupervised method. Combinations (distance vs. metric) for which the Silhouette and Dunn indices reached their maximum values while the Davies-Bouldin index reached its minimum value are marked with an asterisk (*). In each of these cases, the suboptimal number of clusters is indicated (*K* = 3, 4 or 5). The distance-metric combinations for which the criterion above was not met are identified as failures. (**b**) *CD*-index value (in %) vs. number of clusters for the selected seven metrics. Note that the three internal validation indices interact to produce the maximal cohesion-dispersion of the clustering. Thus, the highest of all the *CD*-index values (i.e., *CD* = 100%, Cityblock vs. Correlation) afforded the optimal number of clusters (*K* = 3, marked with an asterisk over the red dashed rectangle). (**c**) Classification applying the SS-SPDF method/algorithm. Note that for the selected recording epoch (1.5 s; at rmPFC), two clusters were significant (cluster 1, 23 spikes; cluster 2, 19 spikes) in their configurations, while the third one was not (cluster 3, 1 outlier). (**d**) At the bottom right are illustrated the principal components analysis (PC1 vs. PC2 plot) and the waveform templates (magenta, brown, and gray profiles) for the resulting clusters. Note that, the action potentials clustering by the SS-SPDF method/algorithm (in **c**) are in perfect correspondence to those spikes (magenta circumferences, brown crosses, and gray square) clustering by principal component analysis (in **d**).

**Figure 6:**
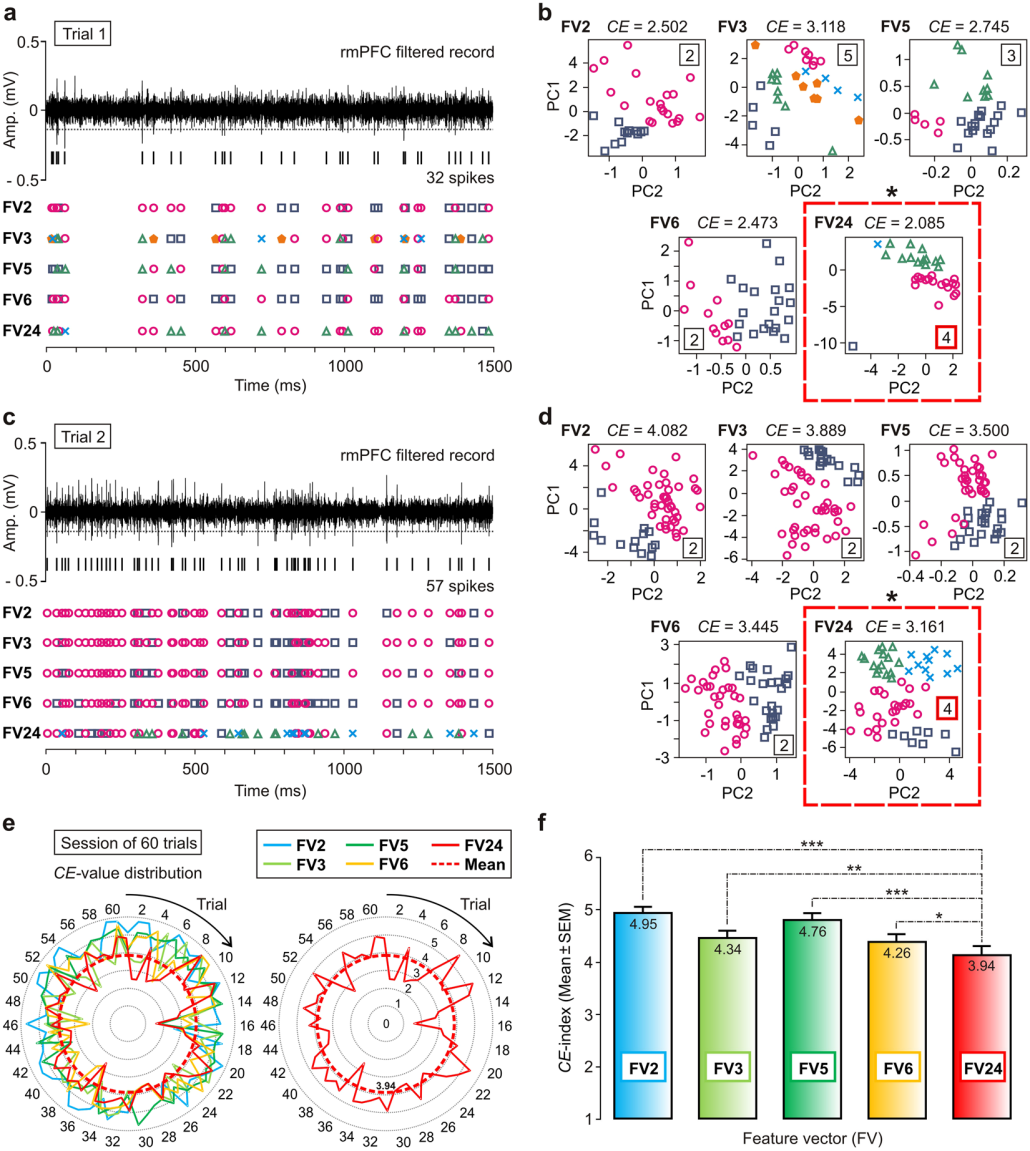
Comparison of the clustering performance between the current SS-SPDF method/algorithm and other methods/algorithms also based on feature extraction. FV2, FV3, FV5, FV6, and FV24 (with 2, 3, 5, 6, and 24 features, respectively) are feature vectors with different features and dimensions (see Table 6). Notice that, the vectors FV2, FV5, FV6 were not subsets of FV24. Only FV3 with three common features is a subset of FV24. (**a**), (**c**) Neuronal activities at the rmPFC corresponding to three representative trials of recordings (epoch of 1.5 s). For each trial, the number of identified spikes (trial 1 in a, 32 spikes; trial 2 in c, 57 spikes) and the different clusters (spike events with the same color symbol) are indicated. (**b**), (**d**) The process for the determination of the optimal clustering among different feature vectors applying the *CE*-index. For all the principal components (PC) analyses (PC1 vs. PC2 plots), the resulting number of clusters is indicated. In addition, the value of the *CE*-index is reported in each panel corresponding to each feature vector (Table 6). For each selected trial, the lowest value of the *CE*-index (trial 1, *CE* = 2.085; trial 2, *CE* = 3.161; applying FV24, with *K* = 4) afforded the optimal clustering among all the classifications (i.e., the one with the lowest value of the *CE*-index, marked with an asterisk over the red dashed rectangle). (**e**) Distribution of the *CE*-index values for the different feature vectors across 60 trials. Once again, the lowest *CE*-index was obtained for FV24, which showed statistically significant differences with respect to the mean values of the *CE*-index obtained by applying the other feature vectors. (**f**) Multiple comparison analyses. For the pairwise comparisons with significant differences between the mean values of the *CE*-index, the significance level is indicated (*P < 0.05; **P < 0.01; ***P < 0.001; see Supplementary Table S4). Data are represented by the Mean ± SEM.

**Figure 7:**
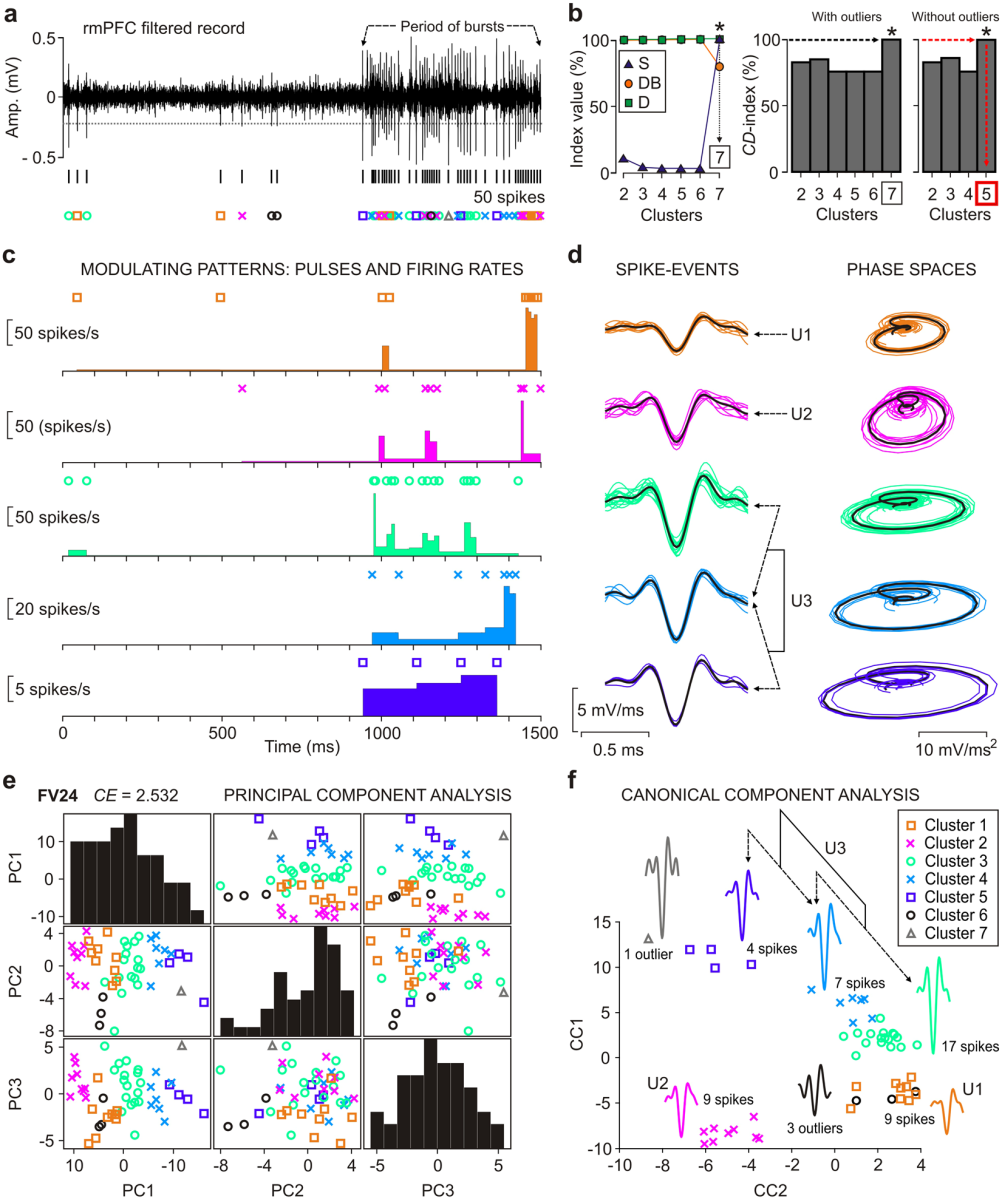
A full enforcement of the SS-SPDF method/algorithm for a single trial of rmPFC recording. (**a**) A representative single trial of rmPFC recording involving single-unit spikes and special spike events (burst activity and spike overlapping). The resulting spike events (*s* = 50) are indicated with different color symbols, one for each cluster of spike events. (**b**) The internal validation indices (S, Silhouette; DB, Davies-Bouldin; and D, Dunn) and the *CD*-index (in %) for the optimal distance-metric combination (Cityblock vs. Jaccard). The optimal number of clusters was *K* = 5 (without outliers) corresponding to the maximum value (100%, red dashed line) of the *CD*-index is indicated. (**c**) Modulating patterns of the identified neurons (five clusters of neurons), including the pulses (spikes with different color symbol in each panel) and the firing rates (in spikes/s). (**d**) Representation of the spike events in both time domain and phase space. (**e**) Principal components (PC1, PC2, and PC3) analysis, indicating the value of the *CE*-index (FV24; *CE* = 2.532) obtained by SS-SPDF method/algorithm. Each normalized histogram in the diagonal represents the number of mixed spike events per band (n = 10) for each principal component. (**f**) Canonical components (CC1 and CC2) analysis. Waveform templates for the resulting clusters (five clusters of spikes, and two clusters of outliers) are showed and their corresponding numbers of spike events are indicated. Also, three different neuronal units (U1, 9 spikes; U2, 9 spikes; and U3, 28 spikes; see panels d and f) are indicated as resulting of the spike sorting analysis for this representative trial of rmPFC recording.

Special emphasis was placed on determining both the optimal number of clusters (employing the internal validation indices and the *CD*-index; see Fig. 5) and the optimal clustering (applying the *CE*-index; see Fig. 6) to assessment the efficiency and reliability of our spike sorting approach on extracellular recordings at the rmPFC of the rabbits (see Figs 5–7). In addition, we examined the computational cost of the SS-SPDF method/algorithm as a function of its execution time at three different time scales of the classical conditioning process (a single trial, a single session, and across learning).

### Determining the Optimal Number of Clusters According to the Proposed Cohesion-Dispersion Index (*CD*-index)

Although the current criterion based on the extreme values of the internal validation indices (i.e., maximum values of Silhouette and Dunn indices in conjunction with the minimum value of Davies-Bouldin index) helps to understand better the results of the classification, we corroborated here that the determination of the optimal number of clusters (*K*), applying this criterion, was dependent on the selected distance-metric combination (see Supplementary Tables S2 and S3, and Fig. S1). Supplementary Fig. S2 shows the values (in %) of the internal validation indices (Silhouette, Dunn, and Davies-Bouldin) for all the combinations (distance vs. metric) after applying *K*-TOPS clustering (*K*-means and template optimization in phase space) on real recordings. For each selected distance, the resulting number of clusters, according to the criterion of the extreme values (*max* and *min*) of the internal validation indices, was different (sqEuclidean, *K* = 5; Cityblock, *K* = 3; Cosine, *K* = 5; Correlation, *K* = 4) and therefore suboptimal, because the goal was to find a single optimal value for the number of clusters.

However, a proper validity measure (*CD*-index) was implemented in this work to obtain the optimal number of clusters among all the distance-metric combinations. The main goal was to make the three internal validation indices interact among themselves in accordance with Eqs (4), (5) and (6) to produce the maximum cohesion-dispersion of the clustering across all the distance-metric combinations (see Methods for details). The performances of both the internal validation indices (Silhouette, Dunn, and Davies-Bouldin, see Fig. 5a) and the proposed *CD*-index (see Fig. 5b) were estimated for all the single trial recordings from rmPFC during the learning process and systematically compared for all the available distance-metric combination. Notice that, in the example of Fig. 5, the *CD*-index always returned definite values (in %) for each distance-metric combination, in contrast with the conventional criterion comprising the extrema of the internal validation indices that left noticeable gaps in the determination of the number of clusters when it was not fulfilled (e.g., see in Fig. 5a the failures in Cityblock vs. sqEuclidean, and Cityblock vs. Cityblock combinations). Finally, the *CD*-index proposed in this work enabled us to successfully find the optimal number of clusters (e.g., *K* = 3 clusters in Fig. 5b) that determined the maximal cohesion-dispersion (100%, marked with an asterisk over the red dashed rectangle in Fig. 5b) of the clustering among all the combinations (distance vs. metric). Note that, for the selected recording epoch (Fig. 5c; rmPFC recording which lasted 1.5 s), two clusters were significant (cluster 1, 23 spikes; cluster 2, 19 spikes) in their configurations, while the other one was not significant (cluster 3, 1 outlier). For this particular example employing the SS-SPDF method/algorithm (Fig. 5c), the number of neurons identified and classified as independent clusters of spikes was n = 2. The resulting template of action potentials for each neuron was also represented as a result of the principal components analysis (see Fig. 5d).

### Determining the Optimal Clustering according to the Proposed Clustering Error Index (*CE*-index)

A review of the current literature on neuronal spike pattern recognition reveals a wide variety of feature extraction methods (Table 1). The proposed SS-SPDF method based on waveform features did not include some of the features proposed by other authors. In this respect, geometric-based features such as area (A)^15^, energy (E)^1,2^, integral transform (IT)^12,15,17,18^, and zero-crossing features (ZCF)^3,21^ were rejected, because these features reached a significant (*P* < 0.05) average correlation-coefficient which value was outside the confidence interval [−0.25 < *r* < 0.25] —i.e., it was likely that these features were not mutually independent (multi-collinearity). For example, we recognize from its mathematical formulations that IT is dependent on the average ZCF, and that E is the square of ZCF. However, the proposed SS-SPDF method included a multi-collinearity verification test. For all the neural events in the recording that exceeded the detection threshold, we construct a matrix of feature vectors of dimension [*N*_*events*_ × *N*_*features*_], that is, a matrix of *N*_*events*_ rows and *N*_*features*_ columns. Then we perform a multi-collinearity verification test to ensure that each column of the same feature for all spike-events has very low correlation with respect to all other columns of features (which are themselves linearly independent according to their algebraic definitions, see Table 3). As a result, we obtained an average correlation coefficient of 0.089 ± 0.02 (mean ± SEM) and *P*-value of 0.26 ± 0.01. This average *P*-value was also obtained as a result of all correlations among columns. Note that the average correlation coefficient was within the confidence interval [−0.25 < *r* < 0.25] and its value was very close to zero (i.e., not-significant correlation; *P*-value > 0.05). In summary, the statistical report also indicates that on average, the 24 physiological parameters of each spike event were independent features —i.e., there is not multi-collinearity among them.

On the other hand, to check the relative performance of clustering among feature vectors with different features and dimensions (FV2, FV3, FV5, FV6, and FV24, with 2, 3, 5, 6, and 24 features, respectively; see Table 6) we implemented the customized *CE*-index (see Methods for details, and Fig. 6). This alternative error index enabled us to verify the misclassification of clustering. For this purpose, we selected two representative recordings (epoch of 1.5 s from rmPFC; Fig. 6a,c) with different adaptive values of their amplitude thresholds (trial 1, *Thr* = −0.1078 mV; and trial 2, *Thr* = −0.1317 mV). The number of identified spikes in each selected trial was 32 (trial 1) or 57 (trial 2). These identified spike events (Fig. 6a,c) were the common start point for the classification processes employing the SS-SPDF method/algorithm and principal component analysis during the representation (Fig. 6b,d).

**Table 6:** Values of the clustering error index (*CE*-index) for feature vectors (FV) with different features and dimensions to check the clustering performance from two representative trials (trials 1 and 2) and from a session of recordings [n = 60 trials, from the rostral-medial prefrontal cortex (rmPFC)].

FV	Feature description/Author	*CE*-index value		*CE* = Mean ± SEM
		Trial 1	Trial 2	Session of 60 trials
FV2	Zero-Crossing Features (ZCF) of the spike. ZC1 (the sum of all the values before zero-crossing) and ZC2 (the sum of values after zero-crossing). Taken from Kamboh & Mason^3^ and Saeed & Kamboh^21^.	*CE* = 2.502 *s* = 32 *d* = 32 × 2 *K* = 2	*CE* = 4.082 *s* = 57 *d* = 57 × 2 *K* = 2	*CE* = 4.951 ± 0.089
FV3	Positive peak [F_14_] of the spike FD, and the positive [F_18_] and negative [F_19_] peaks of the spike second derivative (SD). Taken from Paraskevopoulou *et al*.^19,20^.	*CE* = 3.118 *s* = 32 *d* = 32 × 3 *K* = 5	*CE* = 3.889 *s* = 57 *d* = 57 × 3 *K* = 2	*CE* = 4.340 ± 0.099
FV5	Peak-to-valley amplitude of the spike, waveform duration, negative Integral Transform (nIT), ratio (nIT/maximum peak), and the logarithm of the maximum rise of the spike. Taken from Sonoo & Stalberg^12^.	*CE* = 2.745 *s* = 32 *d* = 32 × 5 *K* = 3	*CE* = 3.500 *s* = 57 *d* = 57 × 5 *K* = 2	*CE* = 4.761 ± 0.088
FV6	Spike peak amplitude, peak roundness (i.e., the spike peak [F_19_] of the SD), the root-mean-square of pre-spike amplitude, the highest repolarization rate, the afterhyperpolarization (i.e., afterspike minimum), and the correlation coefficient between the spike and the reference waveform. Taken from Su *et al*.^22^.	*CE* = 2.473 *s* = 32 *d* = 32 × 6 *K* = 2	*CE* = 3.445 *s* = 57 *d* = 57 × 6 *K* = 2	*CE* = 4.264 ± 0.103
FV24	Description according to the waveform features (shape, phase, and distribution measures) proposed in Table 3 of the current work (SS-SPDF method/algorithm).	*CE* = 2.085 *s* = 32 *d* = 32 × 24 *K* = 4	*CE* = 3.161 *s* = 57 *d* = 57 × 24 *K* = 4	*CE* = 3.935 ± 0.108

*CE*-index values are reported by the Mean ± SEM (standard error of the mean). The total number of spikes (*s*), the dimensionality (*d*) of the method and the number of clusters (*K*) for each trial are also indicated (see Fig. 6). Notice that, the vectors FV2, FV5, FV6 were not subsets of FV24. Only FV3 with three common features (F_14_, F_18_ and F_19_) is a subset of FV24.

The spike-sorting analysis of these trials (Fig. 6a,c) revealed that both the resulting number of clusters and the value of the *CE*-index did not depend on the number of features of the vector. Obviously, the combination of features used in FV24 returns the lowest value of the *CE*-index (trial 1; *CE* = 2.085; *K* = 4; two clusters of neurons and two clusters of outliers), but unlike other methods to evaluate clustering (Tables 1 and 4), the error rate defined here [see Eq. (7) for the *CE*-index] does not depend on the number of extracted features. A lower value of the *CE*-index indicates fewer misclassified and unclassified events, and therefore a better spike-sorting performance for FV24 (*K* = 4) in comparison with the other selected feature vectors (FV2, FV3, FV5, and FV6). Furthermore, the classification employing the feature vector FV24 had the best fit between the value of the *CE*-index and the number of clusters (Table 6), so there was a greater resemblance between the spike waveforms of each cluster and its template. In contrast, the classifications employing the feature vectors FV2 (including the zero-crossing features ZC1 and ZC2) and FV3 (including the common features F_14_, F_18_ and F_19_) reached the two highest inter-trials values (trial 2; FV2, *CE* = 4.082; FV3, *CE* = 3.889) of the *CE*-index, whereas the number of clusters was the lowest (*K* = 2).

In addition, note that for trial 1 (Fig. 6a,b) the classification applying the feature vector FV3 had the worst fit between the value of the *CE*-index (*CE*_max_ = 3.118) and the number of clusters (*K* = 5). Whereas the classification employing FV24 (marked with an asterisk over the red dashed rectangle in Fig. 6b) successfully identified two clusters of neurons with the lowest value of the *CE*-index (trial 1, *CE* = 2.085; cluster 1, 17 spikes; cluster 2, 13 spikes; and two outliers), the classification employing FV3 reported five spurious clusters of neurons, with the highest value of the *CE*-index (trial 1, *CE* = 3.118), indicating that there were misclassified events (Fig. 6b). For trial 2, the lowest value of the *CE*-index (marked with an asterisk over the red dashed rectangle in Fig. 6d) was 3.161 (*K* = 4), also corresponding to FV24. The results for these representative trials indicated that the classification applying a 24D-vector (FV24, with *K* = 4 clusters of neurons) had better clustering performance than the classifications employing the other selected feature vectors (FV2, FV3, FV5, and FV6, all with *K* = 2 clusters of neurons; Table 6).

For each selected feature vector, the values of the *CE*-index for several trials (n = 60, from rmPFC) of a representative recording session (e.g., session C08, during the eyeblink classical conditioning in rabbits) were calculated (Table 6). The range of values for the *CE*-index across the trials and for all the classifications applying the different feature vectors was 1.585 ≤ *CE* ≤ 5.823. Figure 6e shows the distribution of the *CE*-index values across the 60 trials.

For these extended datasets (n = 60 trials), the results also indicated (Fig. 6f) that the lowest mean value of the *CE*-index was obtained for FV24 (*CE* = 3.935 ± 0.108). Furthermore, the multiple comparison analyses revealed that there were statistically significant differences (*P* ≤ 0.03) between the mean values of the *CE*-index for the pairwise comparisons employing FV24 (*CE* = 3.935 ± 0.108) and the other selected feature vectors, whose *CE*-index values (mean ± SEM) are also reported in Table 6. However, there were non-significant differences in the mean values of the *CE*-index for the pairwise comparisons FV2 vs. FV5 (*P* = 0.104) and FV3 vs. FV6 (*P* = 0.646). The reader can see the Supplementary Table S4 for more details about these statistical reports. Interestingly, the results for an extended dataset (n = 60 trials) allowed us to confirm that both the resulting number of clusters and the value of the *CE*-index did not depend on the number of features used in the spike-sorting algorithm. Note that the classification employing FV2 (with two features) and FV5 (with five features) returns similar mean values of the *CE*-index, with the same for the classification applying FV3 (with three features) and FV6 (with six features). Finally, the optimal clustering (i.e., one with the lowest value of the *CE*-index) was obtained employing the 24D-vector of features (FV24) proposed in the present work.

An important advantage of the SS-SPDF method/algorithm proposed here in comparison with some of the ‘gold standard’ approaches [see Table 6: *K*-means clustering + Mahalanobis distance (FV2; taken from Kamboh and Mason^3^; and Saeed and Kamboh^21^); *K*-means clustering + Squared Euclidean metric (FV3; taken from Paraskevopoulou *et al*.^19,20^); Nearest-neighbor Methods + Discriminant Analysis (FV5; taken from Sonoo and Stalberg^12^); *K*-means clustering + Principal Component Analysis (FV6; taken from Su *et al*.^22^)] was obtaining an exhaustive physiological characterization (24 features) of each spike-event in parallel with a substantial improvement of the overall spike sorting performance —i.e., the cohesion-dispersion (*CD*-index) was the highest possible (Fig. 5) when the clustering error (*CE*-index) was the lowest (Fig. 6). Figure 6 represents a proper validation of SS-SPDF method/algorithm showing that during the spike sorting the proposed feature vector (FV24; see Table 3) has more discriminatory power (i.e., an optimal compromise between high cohesion-dispersion and a low clustering error) than the feature vectors (FV2, FV3, FV5, and FV6; see Table 6) proposed by other authors^3,12,19–22^. Notice that, the resulting dimensionality (24D-feature vector per each spike-event from a single electrode, in accordance with the Eq. (2) and the Supplementary Table S5) of the proposed SS-SPDF method/algorithm did not affect either the computation of the clustering indices (*CD*-index and *CE*-index; Figs 5 and 6, respectively) or the total execution time of the algorithm (Fig. 7, Supplementary Table S6 and Fig. S3). Figure 7 shows a full enforcement of our SS-SPDF feature extraction method and of the proposed *K*-TOPS clustering algorithm for a representative trial of rmPFC recording, for which seven clusters were found (*K* = 5 optimal clusters and two clusters of outliers) and the *CE*-index was 2.532 —i.e., the lowest possible value for an optimal clustering according to Eq. (7).

As it is well known, two of the main problems with spike sorting are that if neuron has burst firing and/or the brain moves in relation to electrode then spike waveform will change. Ideally, algorithm should detect such gradual changes in time and classify those spikes as one cluster. Figure 7a shows a representative rmPFC recording obtained while electrode moves (or brain moves in relation to electrode) and neuron has periods of moderate burst. Interestingly, in these two experimental situations were very useful the distribution-based features (F_20_–F_24_; in Table 3) proposed in this study. Note that changes in the amplitudes and waveforms caused direct changes in the probability density function of the spike (e.g., a progressive weight gain of the tails or a transformation towards a non-normal amplitude distribution), which could be exhaustively detected by considering inter-quartile range, kurtosis and asymmetry from the first and second derivatives of the spike. Therefore, combining the proposed SS-SPDF method/algorithm (i.e., twenty-four features from spike derivatives and *K*-means clustering with validity and error indices) with other spike feature extraction methods (principal and canonical component analyses) improved spike sorting performance was achieved, without using Bayesian algorithm^13,49,50^, template matching models in time domain^47,51,52^, wavelets coefficients^53^ or wavelets with superparamagnetic clustering^48^, which usually require computationally advanced training algorithms. Such training algorithms are computational intensive^54–56^, thus slowing down the spike sorting process^16,23^, especially when neuron bursts, the electrode moves, or the brain moves in relation to electrode —i.e., when it is necessary to correctly classify different spike classes that come from a single neuron or to differentiate spatiotemporally overlapping waveforms coming from different neurons.

Although implementation of the alternative training algorithms for the spike sorting of real recordings during eyeblink classical conditioning is admissible, it is assumed that training algorithm should be performed more frequently (i.e., every time the brain moves in relation to electrode and/or the neuron bursts), first with shorter re-training periods across different trials in each particular session, and second with much larger re-training periods across different conditioning sessions; and therefore the implementation becomes complex and difficult. In this work, we proposed the SS-SPDF method/algorithm (i.e., twenty-four features from spike derivatives and *K*-means clustering with validity and error indices; Fig. 7b–d) in combination with the principal (PCA, Fig. 7e) and canonical (CCA, Fig. 7f) component analyses, as an efficient spike sorting approach, which also took into account the aforementioned problems.

Note that, whereas principal component analysis looks for directions of maximum variance, canonical component analysis looks for direction of maximum correlation, therefore, their applications in separate ways are not necessarily the ones offering the best spike classification. However, their joint implementation (combination principal/canonical component analyses) seeks the projections with mixed distributions that indicate the similarity (or not) of clusters in the space of the components, which are very likely the ones offering a more flexible clustering and a better spike classification. At the end of the processing, SS-SPDF method/algorithm always checks the results (Fig. 7a–d), comparing these with those obtained by the combination of principal and canonical component analyses (Fig. 7e,f). Therefore, the proposed SS-SPDF method/algorithm provides the possibility of combining heterogeneous features extracted from the spikes [e.g., derivative-based features in the phase space (including spike shape, phase and distribution features from Table 3), principal component analysis (PCA), and canonical component analysis (CCA)] allowing to take advantage of the strengths of each feature extraction method to achieve better performances. In Fig. 7, spikes from green and cyan clusters (17 and 7 spikes, respectively) were labelled as two spike classes from a single neuron (as it is indicated with a double arrow in Fig. 7d,f) which were separated by the clustering algorithm because electrode was moving (or brain was moving in relation to the electrode) during the record of that particular trial (Fig. 7a).

In addition, if neuron has periods of high and moderate burst rates (Fig. 7a), usually first spike during burst has higher amplitude then others, and challenge again is to detect it and classify those different spikes as coming from a single neuron. Regarding this situation, the proposed SS-SPDF method/algorithm had sufficient discriminatory power to detect and classify spike classes from a single neuron, keeping them grouped within a single flexible group of classes, without violating the assumptions of the neuronal refractory periods and the mean inter-spike interval rates (Fig. 7c). In this way, spikes from cyan and blue clusters (7 and 4 spikes, respectively) were labelled as two spike classes from a single neuron (as it is indicated with a double arrow in Fig. 7d,f). Finally, three different neuronal units (U1, 9 spikes; U2, 9 spikes; and U3, 28 spikes) were detected for this representative trial of rmPFC recording.

### Execution Time of the SS-SPDF Method/Algorithm

We examined the computational cost of the SS-SPDF method/algorithm as a function of its execution time at three different time scales. The first time scale was for a single trial (epoch of 1.5 s, including an inter-stimulus interval) during the eyeblink classical conditioning. Figure 7 shows a full enforcement of our SS-SPDF feature extraction method and of the proposed *K*-TOPS clustering algorithm for a single trial of rmPFC recording, for which the average execution time was 52.282 s. The second time scale was for a single session (n = 60 trials) of recordings, for which the average execution time was 52.2817 min (i.e., 52 min and 17 s). Supplementary Fig. S3 shows the firing modulating rates of the rmPFC neurons obtained after a full implementation of the SS-SPDF method/algorithm for a representative session (60 CS–US trials) of recordings during classical eyeblink conditioning of rabbits. Three different neural activity patterns with significant peaks at three well-defined moments between the start of CS and the end of US (350 ms) were identified after the analysis (including spike sorting) and the exhaustive characterization of the electrophysiological properties of the rmPFC neurons. All this was achieved without using any computationally advanced training algorithms for spike sorting across different trials of a single conditioning session. Finally, the third time scale was for the evolution across the successive conditioning sessions (from C01 to C10), for which the average execution time was of 8.7136 h (i.e., 8 h, 42 min and 49 s). Therefore, the proposed SS-SPDF method/algorithm probably had a linear relationship Ε*τ*(*N*_*trials*_) = *z* × *N*_*trials*_ + *z*_0_ between the execution time (Ε*τ*) and the number of trials (*N*_*trials*_) across the three different time scales of the learning process under study —i.e., the execution time was incremented by z-times when the number of processed trials increased by z.

In addition, we compared the execution times of the computational algorithms employing feature vectors with different features and dimensions (FV2, FV3, FV5, FV6, and FV24). For example, at the asymptotic level of acquisition of this associative learning test (conditioning session C10) there were not statistically significant differences [One-way ANOVA *F*-test; *F*_(4,12,295)_ = 0.456; *P* = 0.768] among the mean values of the execution time employing the different feature vectors. Similar results —i.e., not significant differences (*P* > 0.05) in the mean values of the execution time —were obtained for the other conditioning sessions (from C1 to C9) during the comparison among the different feature vectors (see Supplementary Table S6 for details). Therefore, the total execution time of the proposed method/algorithm using twenty-four features (FV24) was not statistically different to those obtained using fewer features (FV2, FV3, FV5, FV6) across the learning process (from session C1 to C10).

All the computations were developed on a standard personal computer (Inter Core i5-4670 CPU, 3.40 GHz, 4GB-RAM; on Window 8.1 platform) for teaching purposes. We are confident that using divide-and-conquer strategies, the total runtime of our algorithm would be considerably shorter. Another alternative solution to reduce execution times would be to use workstations with greater computational capacity, an optimal combination of CPUs and GPUs, and/or running the algorithms in parallel. Notice that this section was named ‘Execution Time’ and not ‘Computational Complexity’ because the executions in the three different time scales (a single trial, a single session, and across learning) have been implemented using MATLAB (The MathWorks, Natick, MA, USA; version 7.12.0; R2011a) platform, where many high-level functions have been hard-coded. The next step foreseen for analyzing the computational complexity of this algorithm implies its implementation purely on low-level languages (e.g., C/C++), then the execution time will be an acceptable measure of computational complexity. Finally, a coherent combination of all these strategies would allow changing from a linear relationship Ε*τ*(*N*_*trials*_) = *z* × *N*_*trials*_ + *z*_0_ to a more appropriate linear or non-linear relationship (e.g., Ε*τ* = Ε*τ*[*N*_*trials*_log(*N*_*trials*_)]) between the execution time (Ε*τ*) and the number of trials (*N*_*trials*_). In this way our clustering algorithm (*K*-TOPS, two-steps workflow) would report shorter execution times. Also note that, we were not referring to the computational complexity order of *K*-means clustering algorithm (first step alone), which is of type O (*n.k.d.i*) where *n* is the number of *d*-dimensional vectors, *k* is the number of clusters and *i* is the number of iterations needed until convergence. Although this has its limitations, that complexity order and even other higher polynomial orders of complexity can be approachable in practice.

### Summary of the Outcomes

We have demonstrated that the proposed SS-SPDF method/algorithm performs better than other methods^3,12,19–22,24,40–43^ currently used in neurophysiology and also based on feature extraction. First, the prior calculation of the first derivative of the recording for the subsequent selection of the amplitude threshold did not affect the data preprocessing (Fig. 1, left block) and therefore the spike detection step retained the same completeness as for other standard methods —i.e., the optimal performance of both steps (amplitude threshold selection and spike detection, Fig. 2) was guaranteed in this study. Second, the resulting dimensionality (24D-feature vector per each spike-event; see Table 3, Fig. 3 and the Supplementary Table S5) from our SS-SPDF method of feature extraction and the number of distance-metric combinations used during *K*-means clustering (Supplementary Fig. S2) did not affect either the computation of the clustering indices (*CD*-index and *CE*-index or the customized Error Index; see Tables 4–6 and Figs 4–6) or the total execution time of the clustering algorithm (Supplementary Table S6, Fig. S3, and Fig. 7). These parameters (clustering index computation and total execution time) are usually compromised in other classification methods. Third, the spike clustering classification (Fig. 1, right block) improved significantly as a result of the implementation of the proposed validity (*CD*-index) and error (*CE*-index) indices, which allowed us to determine the optimal number of clusters (Fig. 5) and the optimal clustering (Fig. 6), respectively. Fourth, the quantitative comparison with some of the ‘gold standard’ methods (see Table 6 for details) showed that the proposed method (SS-SPDF) improved the overall spike sorting performance —i.e., the cohesion-dispersion (*CD*-index) was the highest possible (Fig. 5) when the clustering error (*CE*-index) was the lowest (Fig. 6). And fifth, the efficiency and reliability of our SS-SPDF method/algorithm was demonstrated in both simulated data (Fig. 4) and real recordings (Figs 5–7) applying an automatic and unsupervised *K*-TOPS clustering algorithm for sorting both the single-unit spikes (by mean of *K*-means on derivative-based feature vectors as main input to the clustering process) and the overlapping waveforms (by mean of TOPS algorithm —i.e., template optimization in phase space). All the aforementioned reasons reaffirm the robustness of the proposed approach for the spike sorting of extracellular recordings from a single electrode and the feasibility of a future expansion of our SS-SPDF method/algorithm to multi-electrode recordings.

## Discussion

The spike-sorting approach is of critical importance, especially in electrophysiology, where the purpose is to understand the functional properties of the neural systems through the analysis of real recordings. The neural information extracted from the spike-sorting process should be useful, not only for the identification and posterior classification of spikes and neurons, but also for the objective characterization of the neural activity events under study^57–60^. In this sense, we have successfully applied an efficient spike-sorting approach that included both the SS-SPDF method of feature extraction and the *K*-TOPS clustering algorithm to detect, identify, and classify spike events (including single-unit spikes and overlapping waveforms) from electrophysiological recordings. In contrast to other methods, also based on feature extraction, the proposed method/algorithm is based on shape, phase, and distribution features of each spike event, which reveal significant information of the neural process under study. The uniqueness of the SS-SPDF method/algorithm is that instead of the reduction of dimensionality adopted in most alternative methods, it carries out spike-sorting analyses based on a dimensionally flexible vector (from 3D to 24D- dimensions) of independent features for each spike event, removing multi-collinearity among the features to simplify the classification process. Furthermore, the classification technique involves the *K*-TOPS clustering algorithm (that is, *K*-means for sorting the single-unit spikes and template optimization in phase space for sorting the overlapping waveforms) with new, useful validity and error indices to verify both the cohesion-dispersion among spike events (*CD*-index) and the misclassification of clustering (*CE*-index), respectively.

The combination of features used in the proposed feature vector (FV24) returns the highest value of the *CD*-index and the lowest value of the *CE*-index, as well as the best fit between them and the optimal number of clusters that determine the maximal cohesion-dispersion of the clustering among all the distance-metric combinations. We consider that the two proposed indices are proper validity and error measures for quantifying the success of a spike-sorting algorithm. In addition, the proposed SS-SPDF method/algorithm ensured that both the resulting number of clusters and the value of the *CE*-index did not depend on the number of features used during the classification. Therefore, the optimal number of clusters and the optimal clustering were strongly depended on the intrinsic physiological properties of the spike events and on the neural process as a whole. These physiological properties were explicitly reflected in the proposed 24D-vector of independent features, allowing a greater resemblance between the spike events of each cluster and its template and the optimal cohesion-dispersion of the clustering —i.e., the proposed twenty-four feature set has more discriminatory power than other sets with fewer features. In addition, we demonstrated that the present SS-SPDF method/algorithm performs better at classifying spikes and neurons and at assessing their modulating properties than other methods also based on feature extraction, —all without using computationally advanced training algorithms for spike sorting along the associative learning process.

Notice that, spike sorting systems based on computationally advanced training algorithms such as artificial neural networks^8,61^, support vector machines^6,54^, template matching models (mostly focused on the variability of the templates in time domain)^6,47,53,54,62–64^, Bayesian algorithms^13,49,50^, wavelets coefficients^53^ or wavelets with superparamagnetic clustering^48^ are not free from inconveniences and often objectionable goals, because all these highly accurate software-based algorithms are strongly focused on high-density microelectrode arrays, overseeing other basic but necessary electrophysiological approaches (e.g., *in vivo* studies using glass micropipettes recordings during associative learning^34–36,38,57,58^, neural oscillations^38^ or clinical disfunction studies from nerve/muscle recordings^59^). A key observation we have made is that the performance of these computationally advanced training algorithms is highly sensitive to the selection of cost functions (e.g., free optimization hyperparameters), transformation factors (e.g., discrete wavelet coefficients) or fundamental components (e.g., independent, principal or canonical components) that are all “abstract” mathematical entities very far-off from any plausible physiological interpretation. All the information that can be used to assign a given spike to its corresponding neuron should be provided essentially by physiological features (not “abstract” mathematical entities) determined from the stereotypical temporal deflections of the action potential (temporal shape of the spike waveform) and by the location of each neuron (spatial shape of the spike waveform). Although the spike sorting systems that employed computationally advanced training algorithms have defined a heuristic which eliminates the need for many manual merges, operator curation is still required, primarily due to non-stationarities in the recordings such as electrode drift. Several strategies combined will reduce this problem^64^. One strategy, particularly appropriate for high-density probes, will be to detect the drifts and spatially shift the raw recordings by the inverse of the drifts. A second strategy could involve modeling more explicitly the variability of the templates as a function of time. However, the strategy that we propose here is to model the variability of the templates in the phase space but only to group the overlapping waveforms because the single-unit spikes can be appropriately classified using *K*-means on derivative-based feature vectors as main input to the clustering process.

In addition, computationally advanced training algorithms are prone to non-optimal local minima^64^ during the optimization process and therefore, must anneal several optimization and regularization parameters, constants, ratios and thresholds at the beginning of the optimization and during training. All these steps are computationally intensive^54–56^ (usually tend to be slow as well, because comparisons of all potential overlapping waveforms in time domain with all template combinations are needed), thus slowing down the spike sorting process^16,23^, especially when the neuron bursts, the electrode moves, or the brain moves in relation to electrode —i.e., when it is necessary to correctly classify different spike classes that come from a single neuron or to differentiate spatiotemporally overlapping waveforms coming from different neurons. It is gradually aggravated when the algorithms are not provided with powerful Linux workstations (e.g., 192 GB RAM and 40 logical processing cores, MountainSort^65^, on UBUNTU package), optimal arrays of several central processing units (CPUs; e.g., Spyking Circus^63^, or KlustaKwik^66,67^, on PYTHON platform), optimal combination of graphical processing units (GPUs) and CPUs (KiloSort^64^, on MATLAB platform) or cloud advanced computing. Interestingly, the confusion matrices (contingency tables that summarize the consistency among different spike sorting of the same data) obtained after the comparison among some of these highly accurate software-based algorithms (Spyking Circus^63^, KiloSort^64^ and MountainSort^65^) revealed that the three algorithms find many of the same units but also highlight a number of clusters where the algorithms produce different results^65^ —i.e., although these three spike sorting systems are sharing similar principles, training algorithms and powerful computational resources, they did not obtain similar performances^65^.

Regarding the manual curation, there would still be some key requirements for Spyking Circus^63^, KiloSort^64^ and KlustaKwik^66,67^. Both KiloSort^64^ and KlustaKwik^66,67^ are typically biased towards producing between two and four times more clusters (or templates) than the expected number of neurons. Spyking Circus^63^ may produce less templates than KiloSort^64^, and thus slightly less manual burden, but still, user will have to review the results manually at this stage. Also, regarding the execution time (not the computational complexity), Spyking Circus^63^, KiloSort^64^, MountainSort^65^ and KlustaKwik^66,67^ (algorithms based on template-matching in time domain) are several orders of magnitude faster than standard mixture-of-Gaussians fitting^67^. Nevertheless, when these software-based systems are running on large datasets, it can take hours or even days to complete on a standard single-processor machine^67^, depending on the duration of the neural event. However, notice that in the situation of a low-density electrode array and limited computational resources, these software-based systems (Spyking Circus^63^, KiloSort^64^, MountainSort^65^ and KlustaKwik^66,67^) solve the problem of overlapping waveforms in different ways, but always using template-matching in the time domain, which leads to different spike sorting performances^65,67^. Furthermore, with less channels and less density, the resolution of spatiotemporally overlapping waveforms (i.e., the assignment of each of them to a single-unit cluster) becomes less tractable and, at the same time, less interesting from a mere computational point of view^63–67^; but not from an electrophysiological framework^34–36,38,57,58^.

In this work, we use a proper combination of a dimensionally flexible vector of derivative-based features (including spike shape, phase and distribution features) from each spike event and *K*-TOPS clustering (*K*-means and template optimization in phase space) with new validity (*CD*-index) and error (*CE*-index) indices, which shows high accuracy for all the neural datasets we have tested (simulated data and rmPFC extracellular recordings). All the validation tests were developed on a standard single-processor machine without using any computationally advanced training algorithms for spike sorting across different trials of a single conditioning session and along successive sessions during classical eyeblink conditioning^38^. The SS-SPDF method/algorithm we have described here, constitutes both a practical platform for new methods based on spike feature extraction (in both time domain and phase space) with physiological relevance and also a framework from which to develop solutions based on spike waveform (temporal and spatial shapes) for present and future generations of highly accurate spike-sorting systems for neural recordings from high-density microelectrode arrays. Finally, the proposed SS-SPDF method/algorithm has been implemented as an easy-to-use software platform (called *VISSOR*, see the section Spike-Sorting System/Software Overview in Methods), which will further extend its future usability and impact in the neural spike sorting field.

## Methods

### Simulated Data

To evaluate the performance of our method/algorithm and to compare it with other methods/algorithms we employed a simulated data. The simulated data has some advantages over the real data for evaluating the performance of the algorithm, because it provides known solutions under different conditions, such as total number of spike events (including multi-unit and overlapping waveforms), potential templates, firing time, firing rate and so on. Simulated data (sampling frequency of 44 kHz and duration of 180 s) were ceded by the Neuro-Heuristic Research Group at University of Lausanne, Switzerland^40–42^. The simulated spike train was designed by using background noise and three spike templates (*T*1, *T*2 and *T*3, see Fig. 4) generated in a similar way to those reported by other authors^24,38,40–42^. Notice that, whereas templates *T*1 and *T*2 had a triphasic time course, template *T*3 was only biphasic. Consequently, to make sure that the 3 shapes obtained from templates *T*1, *T*2 and *T*3 are not too specific and for properly assessing the sorting capabilities of the *K*-TOPS method/algorithm, we randomly injected spikes with comparable peak amplitudes from templates *T*1 and *T*2, but with other different features from FV24 feature vector. This strategy was similar to that originally used by Letelier & Weber^24^ and then applied by other authors^38,40–42^.

In this study, we added 2700 instances of each template randomly to the background noise, avoiding template overlapping —i.e., 8100 single-unit spikes. The resulting spike train mimicked three neurons firing independently at an average rate of 15 spikes/s and contained 81 overlapping waveforms (with a delay of less than 2 ms between peaks), among which 45 waveforms were the overlapping of spikes from two templates and 36 waveforms were the overlapping of spikes from three templates. In order to generate the overlapping waveforms, first, for each single-unit template combination, the selected spikes were shifted relative to each other, and then were added together point by point to create a superposition waveform corresponding to each shift. The next step was to add the simulated overlapping waveforms in the noise signal. Because of our perfect knowledge of the point in the noise signal where each selected spike was added, superposition events always generated separate profiles, and no spikes went undetected. The procedure of defining the single unit templates, generating the overlapping waveforms (by combinations of two or three templates) and adding them in different specific points of the noise signal was repeated five times (from D_1 to D_5 simulated datasets) and the results from five runs were reported in Table 4.

### Experimental Data

To evaluate the performance of the SS-SPDF method/algorithm and to compare it with other methods/algorithms also based in feature extraction we employed a real experimental data. Electrophysiological recordings used in the present study were collected from adult male rabbits (New Zealand white albino), obtained from an authorized supplier (Isoquimen, Barcelona, Spain). All experimental procedures were performed in accordance with European Union (2010/63/EU) guidelines and Spanish (BOE 34/11370-421, 2013) regulations for the use of laboratory animals in chronic experiments. Experimental protocols were also approved by the local University Ethics Committee (Pablo de Olavide University of Seville). The surgical preparation, and other experimental details, have been described elsewhere^38,68,69^. Neuronal electrical activity recorded in the rmPFC area was performed with glass micropipettes filled with 2 M NaCl (3–6 MΩ of resistance), with the help of a NEX-1 preamplifier (Biomedical Engineering). These recordings were analogically filtered in a bandwidth of 1 Hz to 10 kHz by a AC/DC differential amplifier (A-M System 3000) that is designed for low-noise recordings from excitable tissues. It is intended for extracellular recordings and/or stimulating in conjunction with microelectrodes. The recording area was approached with the help of stereotaxic coordinates^70,71^. The recording site was changed in the horizontal plane in steps of 0.1 mm until a suitable unit was recorded and identified^38,58,68,69^. The unitary activity recorded in the rmPFC was acquired on-line through an 8-channel analog-to-digital converter (1401-plus; CED) where the neural recordings were digitized and transferred to a computer for quantitative off-line analysis. Data were sampled at 25 kHz and with an amplitude resolution of 12 bits in order to make a suitable recognition of the spike waveform.

### Signal Preprocessing Overview

The first step in the preprocessing was signal filtering. In this work, extracellular rmPFC recordings were sampled at 25 kHz (Fig. 2a) and digitally filtered (Fig. 2b) with a bandpass (range from 450 to 2050 Hz) FIR filter^23,54^ —i.e., including spikes whose durations (absolute refractory periods) were ranging from 0.5 to 2.2 ms. For selecting this frequency range, we developed an algorithm based on regular differentiations of a kernel function^49^ generated by a Gaussian curve with the same mean and standard deviation as the raw electrophysiological recording. This strategy is meant to ensure that the optimal power spectrum is included in the interest range. Thus, the maximum frequency (2050 Hz) of the range was in correspondence with the minimum spike duration (0.5 ms), and the minimum frequency (450 Hz) of the range with the maximum spike duration (2.2 ms). This optimal frequency range (from 450 to 2050 Hz) for filtering the rmPFC recordings allowed us to remove low-frequency fluctuations, such as the local field potential (LFP) and other artifacts. Afterwards, the regular differentiations (first-order and second-order derivatives) of the filtered recordings were calculated to stabilize the resulting recording^16^.

The subsequent preprocessing step was the selection of the amplitude threshold (horizontal dotted line in Fig. 2b) for the preliminary spike detection and identification (Fig. 2c). Note that if the amplitude threshold is selected automatically or it depends on a choice of the investigator, multiple thresholds could be chosen, as there are multiple neurons with similar-shaped waveforms but different amplitudes, and the spike amplitude distribution could be multimodal. To avoid this problem, we performed spike detection applying an adaptive threshold that fitted the data points of the extracellular recording once the possible artifacts had been removed and the segments contaminated by them rejected. The artifacts of the extracellular recordings were atypical waveforms or oscillations caused by an increase in the connection resistance between the recording electrode and the tissue impedance. They were evident because their peak-to-valley amplitudes were significant (*P* < 0.001) with respect to the mean peak-to-valley amplitudes of the recording. In this work, the automatic adaptive threshold (*Thr*) was set to three times the median absolute deviation of the first-order derivative $\dot{V}(t)$ of the band-pass filtered signal [see Eq. (1)], in order to exclude noise that could interfere with template identification.

1 $$Thr=\pm \,q\cdot {\sigma }_{n};\,{\sigma }_{n}=median\{\frac{|\dot{V}(t)|}{0.6745}\}$$

The value of the constant *q* was typically between 3 and 5. The denominator in the formula above is the inverse of the cumulative distribution function for the standard normal distribution evaluated at 0.75. In general, the median absolute deviation *σ*_*n*_ of the filtered recording is an estimate of the standard deviation of the background noise^5,48,72^. Several authors^19,20,40–42^ have applied this derivative-based criterion [see Eq. (1)] for determining the adaptive threshold. Instead of the signal itself, let us consider the first-order derivative $\dot{V}(t)$ of the filtered recording —i.e., the occurrence of a spike is detected whenever the value of the first derivative of the filtered recording exceeded this threshold. Selection of $\dot{V}(t)$ instead the signal itself is also supported by mathematical demonstration^40^. Threshold (*Thr*) obtained from the first derivative can also be applied for spike detection directly from the filtered recording. However, in practice, during real-time spike recognition, the threshold could be adjusted explicitly to achieve better performance according to the experience of the user.

Finally, all extracted spike events were aligned (Fig. 2d, Left) based on their negative peak positions —a step that improves the classification process, and the phase-space portraits^40^ of all the aligned spikes were reconstructed (Fig. 2d, Right). For each detected spike, twenty-four physiological features (Tables 2 and 3) were assembled for further processing. Details of other steps are described in the following subsections.

### Feature Extraction

The feature extraction process is a critical step in spike-sorting methods after detection of the spike in an actual recording. The extraction of the features is based on the spike waveforms^13,37,73^, discrete wavelet transform^5,24,48^, or *K*-means clustering^39,52,53^ for the classification step. Although certain studies have shown that wavelet-based feature extraction potentially outperforms other feature extraction methods based on waveforms^74,75^ or principal components^76,77^, this cannot be generalized to all possible neural datasets^78^. Therefore, an a priori exclusion of features based on waveform or principal components can limit the ability to discriminate between certain spike events^2,78^. In fact, this is also true for the phase and distribution features (see Table 3 for details) that have been little utilized in most cases, but it should not be considered a general rule, because these features can be very appropriate during the spike-classification of real recordings. For example, the maximum spike amplitude (F_14_) of the first derivative of the action potential, or the interquartile metrics (F_20_ and F_21_) of the spike waveform can return considerable highlight differences among spikes and classify them better than other specific coefficients or components separately.

In contrast to other methods also based on feature extraction (see Table 1), the method presented here involves several possible waveform-based features that included shape (features from spike waveform first derivative in time domain), phase (features from spike trajectory in the phase-space: first derivative vs. second derivative), and distribution (features from spike amplitude distribution function for both the first and second derivatives) measures of each spike event. Note that spike clustering is more efficient in a greater dimension, where more information on the spike waveforms is available. The difficulty arising from the high dimensionality of the data space should be mitigated by eliminating redundant data information^79^ in two ways: operating with the least amount of wavelet coefficients^5,48^ or principal components^2,76–78^ as possible, or simply working with as many independent waveform-based features as possible. In this work, we selected the independent features (n = 24, see Table 3) that best described and separated the neuronal spikes, removing the multi-collinearity to simplify the classification process. The independent features (F_1_–F_24_) proposed here ensure that the feature vector in a 24D-space (R^24^) does not hold redundant information, and thereby remove the need to further reduce the dimensionality following the standard way of the principal component analyses. Furthermore, these independent features (see Fig. 3 for details) can be quickly and easily calculated and represent no imminent threat to the computational cost and complexity of the algorithm. In summary, we proposed 21 new features (see Table 3) in addition to the three common features (F_14_, F_18_ and F_19_) used by other authors (see Table 1).

Finally, a key observation we have made is that depending on the electrode array density and on the number of recording sites, our SS-SPDF method/algorithm can optimize the feature vector dimensionality —i.e., we offer a dimensionally flexible vector (from 3D to 24D- dimensions) where each feature subset of this vector was an appropriate independent-feature vector (see algebraic definition in Table 3). From a mathematical point of view, the 24 features proposed here (or any optimal subset of them) are principal components in an orthogonal space of representation. A weight function (0 ≤ *W*(*f*_*i*_) ≤ 1) distributes the actual contribution of each feature of the 24D-vector to spikes/neurons clustering. Values of *W*(*f*_*i*_) close to 1 indicate highly-significant contribution, while values close to zero determine a very low contribution. The proposed weight function *W*(*f*_*i*_) distributes the feature contributions, optimizes the feature vector and also reduces dimensionality, rejecting those features that have less weight (or very low contribution). For the optimization of the feature vector dimensionality as a function of the number of electrodes we used the criterion:

2 $$(\sum _{i=1}^{{N}_{features}}\,W({f}_{i})/{N}_{features})\times ({N}_{electrodes}/({N}_{electrodes}+{N}_{features})) < C$$

In Eq. (2), $\sum _{i=1}^{{N}_{features}}\,W({f}_{i})$ is the sum of weights of all the features, *N*_*features*_ and *N*_*electrodes*_ are the numbers of features and electrodes, respectively; and *C* is a parameter which values must be carefully chosen by the user. The parameter *C* = 1 when *W*(*f*_*i*_) = 1 and *N*_*electrodes*_ ≫ *N*_*features*_. Supplementary Table S5 summarizes the *C*-values for some supposed electrode arrays. For example, for a 32-channel electrode array (2^5^ electrodes) and selecting *C* < 0.5, the feature vector dimensionality must be reduced to 18 features (those having greater weight) and this selection would ensure a proper spike sorting performance. However, selecting *C* < 0.25 the feature vector dimensionality must be reduced to the 7 features with greatest weight to successfully sort the neural events from the same 32-channel electrode array. In summary, with *C* < 0.5 and *C* < 0.25 the following relations between *N*_*electrodes*_ and *N*_*features*_ can be obtained. For *C* < 0.5 (see yellow cells in Supplementary Table S5): [from 2^0^ to 2^4^ electrodes, 24 features]; [2^5^ electrodes, 18 features]; [2^6^ electrodes, 14 features]; [2^7^ electrodes, 13 features]; [from 2^8^ to 2^10^ electrodes, 12 features]; and [from 2^11^ to 2^19^ electrodes, 11 features]. For *C* < 0.25 (see green cells in Supplementary Table S5): [from 2^0^ to 2^2^ electrodes, 24 features]; [2^3^ electrodes, 22 features]; [2^4^ electrodes, 9 features]; [2^5^ electrodes, 7 features]; [from 2^6^ to 2^8^ electrodes, 6 features]; and [from 2^9^ to 2^19^ electrodes, 5 features]. The advantage of this procedure is that the resulting feature vector (optimal subset of features) preserves features susceptible to an appropriate physiological interpretation, —i.e., they are not “abstract” mathematical entities (components, coefficients, factors, or any other hyperparameter). Based on this assessment procedure of the feature weights, it is also possible to identify the features that explicitly discriminate between two different spike shapes, which is advantageous in neural recordings that include more than just two signal shapes (e.g., different types of pyramidal neurons and interneurons).

#### Shape-based features

After the six fundamental points (P_1_–P_6_, see Table 2) of the spike waveform in the time domain of the first derivative of the action potential (see Fig. 3a) are fixed, the feature extraction algorithm returns 11 shape-based features. These new features (F_1_–F_11_, see Table 3) were self-explanatory and provided essential information of the spike event during the spike classification of real neural data (e.g., rmPFC extracellular recordings). Note that in this work we have successfully used some shape-based features (F_1_, F_2_, F_4_, F_5_, F_6_, F_8_, and F_11_, from the spike first derivative), with similar algebraic definitions to those used by other authors^1–4,12–14,22,23^, during the feature extraction directly from the spike and not from its first derivative.

#### Phase-based features

In this step, the first- and second-order derivatives^37,40,42^ of the filtered recording were calculated. Eight features (F_12_ – F_19_; see Table 3) of the spike trajectory in the phase space [second derivative (SD) vs. first derivative (FD), see Fig. 3b] were extracted. Five of these phase-based features were proposed as new features in this work: amplitudes F_12_, F_13_, F_15_, and F_16_ of the FD of the action potential corresponding to the points P_1_, P_3_, P_5_, and P_6_, respectively; and the amplitude F_17_ of the SD of the action potential corresponding to P_1_. These new phase-based features (F_12_, F_13_, F_15_, F_16_, and F_17_) allowed us to preserve the independence in this set of features and resolve better the overlapping problem of the spikes. The remaining phase-based features (F_14_, F_18_, and F_19_) were common features used with good results by some of the authors^16,19,20,22,23^ cited in Table 1.

#### Distribution-based features

One of the most important contributions of this work is the extraction of features directly related to statistical distribution measures of each spike, considering the sample of amplitude values from the FD (or SD) of each action potential. Using basic interquartile ranges, kurtosis coefficient, and Fisher asymmetry measures of the FD and SD, we extracted five distribution-based features (F_20_–F_24_; see Table 3) from each spike. For the calculation of these distribution measures, two tests were performed: (1) selecting only the points of the positive and negative components of the spike waveform; and (2) selecting all the points of the spike waveform. The best results were obtained with all the points of the spike waveform. In particular, the kurtosis coefficient^80^ was calculated from both FD and SD, obtaining similar results; therefore, the feature F_22_ was extracted from the FD of the action potential.

Note that the interquartile range F_20_ (or F_21_) is the difference between the 75^th^ percentile and the 25^th^ percentile of the amplitude values from the FD (or SD) of each action potential, and is the most significant basic robust measure of scale to quantify the statistical dispersion of the amplitude values of a spike. Thus, spikes of different sizes will have different interquartile ranges. In the same way, the kurtosis coefficient (F_22_) measures the critical changes in the tail weights and peakedness of the probability density function for the amplitudes of each spike event. In this situation, kurtosis largely reflects tail behavior of the amplitude distribution—i.e., the distribution will have a high kurtosis coefficient if there is a concentration of values near its tails (heavy-tailed distribution). Therefore, the presence of action potentials with large positive and negative values leads to locally heavy tails of the amplitude distribution. Finally, the features F_23_ and F_24_ (Fisher asymmetries) measure the relative deviation of the distribution mode with respect to mean value of amplitude distribution of the spike FD (see Fig. 3c). For a unimodal distribution, a negative asymmetry coefficient indicates that the tail on the left side of the probability density function is longer or fatter than that on the right side—it does not distinguish these two kinds of shape, but it was a reliable geometric measurement. Conversely, positive asymmetry coefficient indicates that the tail on the right side of the probability density function is longer or fatter than that on the left side.

Some of the distribution-based features may be useful in detecting wavelet coefficients related to neural bursts of raw muscle sympathetic nerve activity (MSNA neurogram). For example, local kurtosis was an optimal method to separate pure noise wavelet coefficients from those associated with MSNA bursts^59^. In general, during periods of moderate and high burst rates, the tails of the probability density function become progressively heavier and the amplitude distribution is non-normal, but during periods of neural silence (noise-related epochs), the distribution is nearly normal (or Gaussian). However, to our knowledge, the distribution-based features have not previously been applied to spike sorting from extracellular recordings of neuronal activity. As supported by a previous study^38^, the results of this work shows that the new features (F_20_–F_24_) were very powerful during the spike-classification from the rmPFC extracellular activity.

### Clustering Algorithm: *K-*means and Template Optimization in Phase Space (*K*-TOPS)

Although the clustering algorithm is a separate subblock (see Fig. 1), the performance of the clustering classification is also dependent on the quality of the preceding feature extraction. In the same way, the overall spike-sorting performance is additionally dependent on the spike classification. The unsupervised clustering algorithm was the most complex part of the spike-sorting process (Supplementary Appendices S1–S5 and Figs S1–S3). We applied *K*-means clustering^39,81^ for sorting the single-unit spikes and for identifying the overlapping waveforms, which requires *K* inputs and is based on distance metrics calculation. In this work, the *K*-means clustering method was unsupervised (i.e., it did not depend on a number *K* of clusters introduced by the researcher) and completely automatic (i.e., the input *K* was iterated from two to root-square of the number of detected spikes). Note that the maximum number of clusters (*K*_max_) that can be present in a neural dataset having *s* spikes should not exceed the value $\sqrt{s}$. This value of *K*_max_ is considered to be a rule of thumb in the clustering literature^81–83^. The basic idea was, given an initial, but not optimal clustering, to relocate each point to its new nearest center —that is, to update the clustering centers by calculating the mean of the member points—and to repeat the relocating-and-updating process until convergence criteria (such as the predefined number of iterations and/or difference in the value of the distortion function) are satisfied. In this way, we clustered the single-unit spikes in different clusters, and largest and densest clusters that showed appropriate cohesion-dispersion inside-and-among them were selected as potential single-unit clusters. After the single-unit clusters were chosen, the remaining clusters consisted of overlapping waveforms, multi-unit waveforms with very small amplitudes, stimulus artifact or simply outliers. The less representative clusters with small amplitude due to background noise and the non-representative events due to artifacts were automatically removed following the criteria previously proposed by other authors^40,41^. The remained events clearly identified as outliers were also eliminated.

**Figure 8:**
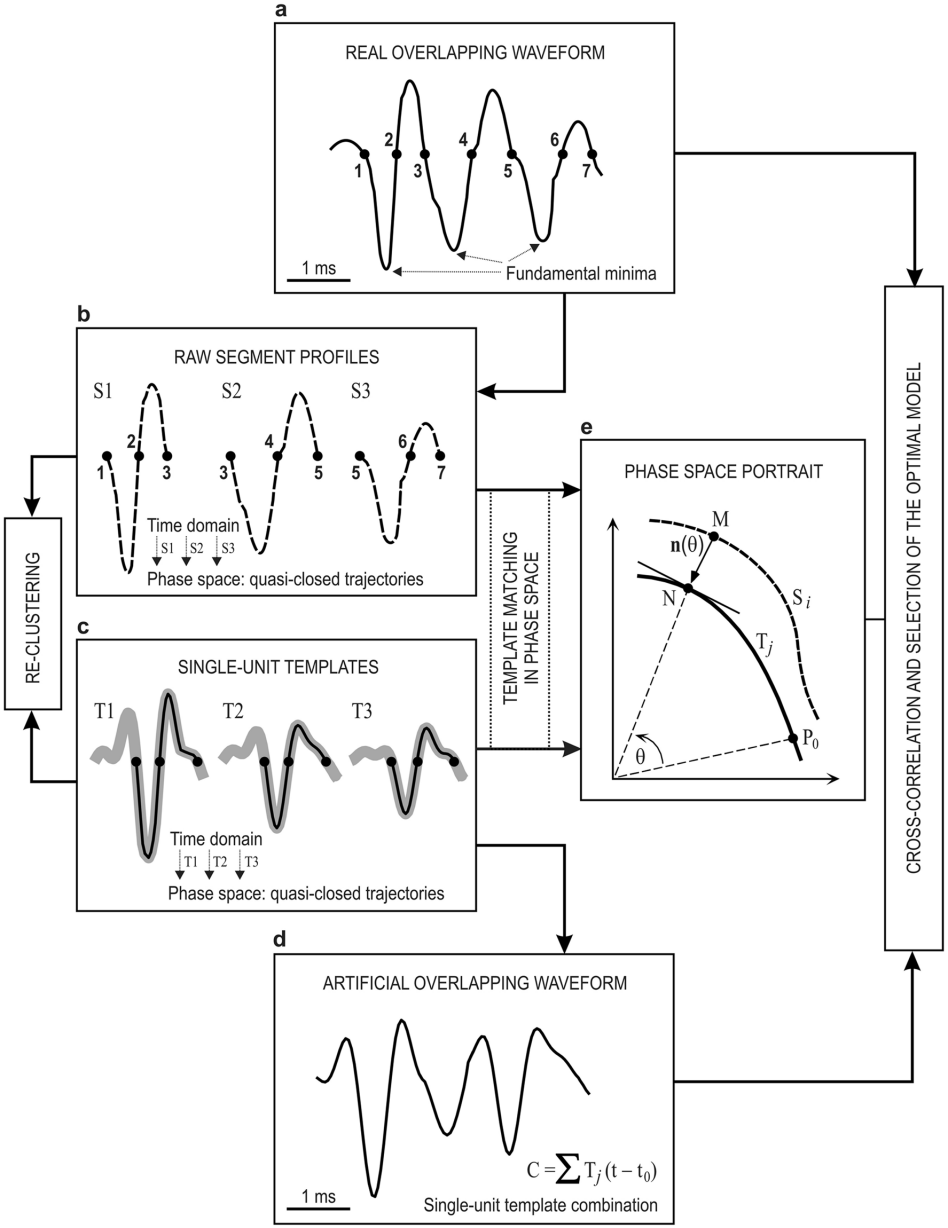
Block diagram of the overlapping waveform separation algorithm using template optimization in phase space (TOPS clustering algorithm, second step). (**a**) Real overlapping waveform. The arrows indicate the fundamental minima and the different points (from 1 to 7) are the zeros. (**b**) Raw segment profiles (dashed traces) extracted from the real overlapping waveform according to the criterion for the fundamental minima. Each segment in time domain corresponds to a quasi-closed trajectory in the phase space. (**c**) Single-unit templates (gray traces) obtained from *K*-means clustering (first step). Here are also indicated the extracted segments in time domain (black traces) corresponding to quasi-closed trajectories in the phase space. (**d**) Artificial overlapping waveform obtained by linear superposition of the single-unit templates with time shift sequences smaller than 2 ms between peaks. (**e**) Phase space portrait and the new variables *θ* (phase) and *n*(*θ*) (normal deviation) that were introduced to describe the trajectory of the analyzed signal (i.e., the reconstructed raw segment profile *S*_*i*_ in the phase space, see dashed trace). The length of the vector |*n*(*θ*)| corresponds to the minimal distance between the signal *S*_*i*_ and the limit trajectory *T*_*j*_ (i.e., one reconstructed single-unit template *T*_*j*_ in the phase space, see black trace). *P*_0_, *M* and *N* are reference points of the trajectories in the phase space. Finally, to find the artificial overlapping waveform which resembles the real overlapping waveform as good as possible, the cross-correlation is applied between them, for all the combinations.

In addition, we have implemented the template optimization in phase space for sorting the identified overlapping waveforms (see Fig. 8a–e). This approach of template optimization in phase space was based on the same assumption as previous spike sorting systems^40–43^, which assumed that the activity of each neuron is described by its own dynamic in the phase space. They carry out template matching in phase space for sorting the single-unit spikes by comparisons of all spikes with all the available single-unit templates. However, in the present work we have applied “template matching”, or as we have alternatively called it, “template optimization” in phase space, for assigning each overlapping waveform obtained from real recording to its corresponding single-unit cluster by comparisons of all potential overlapping waveforms with all single-unit template combinations. Single-unit template combinations allowed us to generate artificial overlapping waveforms by linear template superpositions with relative time shift between them. The next step was to group both real (experimentally identified) and artificial (computationally generated) overlapping waveforms, and then re-sorting this new set of overlapping waveforms by measuring the distances between their trajectories in phase space, with special emphasis in the relative distance between their corresponding fundamental minima. This procedure enabled us to obtain the best combination of single-unit templates for each real overlapping waveform. Therefore, it was necessary to determine the number of points of the overlapping waveform where its first derivative was zero and its second derivative was positive simultaneously (i.e., the minima of the overlapping waveform in time domain). This computation was checked by systematic examination of the phase space of each overlapping waveform.

The assignment of each real overlapping waveform to its corresponding single-unit cluster was made based on the number of fundamental minima of real overlapping waveform that exceeded the detection threshold and on the effect that each single-unit template had on the artificial overlapping waveform that best matched (in the sense of Pearson correlation coefficient) the real overlapping waveform. Because of our perfect knowledge of the contribution of each single-unit template to the generation of each artificial overlapping waveform, as well as, of the number of fundamental minima of both real and artificial overlapping waveforms, the proposed algorithm of template optimization in phase space always returns an assignment solution. This algorithm in phase space also belongs to the wide class of template matching methods for spike sorting and the mathematical aspects were described in detail in Aksenova *et al*.^40^, Asai *et al*.^41^ and Chivirova *et al*.^42^. In this section we present only a short description of the TOPS step of the proposed *K*-TOPS clustering algorithm, in accordance with the block diagram of the overlapping waveform separation procedure (Fig. 8a–e). Notice that our approach was based on the inverse method of nonlinear oscillation theory^40–43^, for which an ideal undisturbed spike is represented as a closed self-oscillating trajectory in the phase space known as stable limit cycle. Therefore, we have assumed that in the phase space (spike first derivative vs. its second derivative) the external trajectory that is attributed to the main part of the spike event contains at least one point where the spike first derivative is zero and spike second derivative is positive simultaneously (i.e., at least one minimum of the spike in time domain).

If only one fundamental minimum in the real overlapping waveform was detected, the real overlapping waveform would be assigned to all available single-unit clusters. This assignment was made based on the assumption that all single units fired very close to each other in time and that it was possible that their single-unit spikes occurred quasi-simultaneously. As a result, the detected overlapping waveform, which was likely generated as a mere amplitude superposition of the single-unit spikes, only contained one minimum. Notice that this amplitude superposition occurs without clearly reflecting the potential contribution of each single-unit spike to the overlapping spike, so it was classified as an overlapping waveform, remaining out of all single-unit clusters, as a result of the first classification step. However, the synchronization of the discharges from the single units clearly suggests assigning one spike to each single-unit cluster.

If more than one fundamental minimum were detected in the real overlapping waveform (e.g., see Fig. 8a), then the problem of assigning an overlapping waveform to one of the single-unit clusters became the problem of assigning each negative waveform component containing one minimum of the real overlapping waveform to its corresponding single-unit cluster. In this way, around each overlapping waveform minimum exceeding the detection threshold, two subsets of points of the overlapping waveform were selected (forwards and backwards from the minimum) until completing a closed (or quasi-closed) trajectory in phase space. These outer closed trajectories preserve essential characteristics of the spike events in phase space and allowed us to reconstruct typical waveforms in time domain, which were representing raw segments of the real overlapping waveforms. All these rational segments (see Fig. 8b) were automatically extracted and systematically compared with all single-unit templates (see Fig. 8c) in order to assign each rational segment containing one minimum of the real overlapping waveform to its corresponding single-unit cluster. This assignment was made based on the assumption that single units fired relatively far apart in time from each other and that it was possible that a waveform (time-amplitude) superposition of the single-unit spikes occurred. As a result, the detected overlapping waveform contained more than one fundamental minimum and both real overlapping waveform and the segments extracted of it (i.e., the negative waveform component containing one minimum) had minor waveform differences when compared to the single unit spikes. This situation also contemplates the possibility that one of the extracted segments (each containing one minimum) contributes very little to the real overlapping waveform with respect to the remaining rational segments and therefore they could not be assigned to any single-unit cluster. In this way, the remaining rational segments of the real overlapping waveform should be assigned to the single-unit clusters which templates mainly contributed to the artificial overlapping waveform that best matched the real overlapping waveform. This procedure ensures the negative waveform components containing the remaining fundamental minima that best matched the single-unit templates to be assigned to their corresponding single-unit clusters.

This approach is distinct from that developed by other authors^40–43^ because the classification proposed by them did not handle spike overlapping. The reconstructed phase space portrait (RPSP) from the major portrait radius (MPR)^43^ of the overlapping waveform as a whole (without decomposition by segments according to the number of minima) still preserves essential waveform characteristics and major trajectory but did not solve the problem of spike overlapping.

Finally, applying the sequence of first, *K*-means clustering for sorting the single-unit spikes (assigning each of them to its corresponding single-unit cluster) and for identifying the overlapping waveforms, and then, template optimization in phase space for sorting the subsets of identified overlapping waveforms (also assigning each of them to its corresponding single-unit cluster), we achieved an efficient clustering algorithm (*K*-TOPS) that considered the problem raised by the relative time-amplitude variations of the spike waveforms in two scenarios: (1) different spike waveforms generated from a single neuron caused by electrode movement or bursting neurons and (2) overlapping waveforms resulting from the near simultaneous firing of multiple neurons.

#### CD-index: clustering validity measure

Many indices have been developed and compared for determining clustering validity^83–90^. As the goal of clustering is to make objects within the same cluster similar, and objects in different clusters distinct, internal validation index measures are often based on two criteria: [1] compactness (which measures how closely related the objects in a cluster are) and [2] separation (which measures how distinct or well-separated a cluster is from other clusters). The goal was to find the best partition and the optimal number of clusters by using internal validation measures: Silhouette (Supplementary Appendix S2), Davies-Bouldin (Supplementary Appendix S3), and Dunn (Supplementary Appendix S4) indices.

The Silhouette index validates the clustering performance based on the pairwise difference of between- and within-cluster distances. Dunn’s index uses the minimum pairwise distance between objects in different clusters as the intercluster separation and the maximum diameter among all clusters as the intracluster compactness. The optimal number of clusters is determined by maximizing the values of both Silhouette and Dunn indices. However, the Davies-Bouldin index is calculated as follows: for each cluster *C*, the similarities between *C* and all other clusters are computed, and the highest value is assigned to *C* as its cluster similarity. Then, the Davies-Bouldin index can be obtained by averaging all the cluster similarities. The smaller the Davies-Bouldin index value, the better the clustering result. In other words, when the clusters present their biggest difference, the best partition is achieved. For computation, this work contemplated four different measures of distance (sqEuclidean, Cityblock, Cosine, and Correlation; see Supplementary Table S2) between clusters and seven clustering metrics (sqEuclidean, Euclidean, Cityblock, Cosine, Correlation, Hamming, and Jaccard; see Supplementary Table S3). Thereby, we obtained twenty-eight (4 distances x 7 metrics) different values for each internal validation index to compare among them (see Supplementary Fig. S1), and to obtain both the best partition and the optimal number of clusters (see Supplementary Fig. S2 and Fig. 5 in the main text).

For objectively evaluating the performance of the clustering by the *K*-TOPS algorithm, in conjunction with the three internal validation indices, a unified validity index for determining the cohesion and dispersion of the clustering was proposed. This cohesion-dispersion index (*CD*-index) for each clustering was calculated using the probabilities (*p*_*S*_,*p*_*DB*_,*p*_*D*_) of the three internal validation indices (Silhouette, *S*; Davies-Bouldin, *DB*; and Dunn, *D*) and their respective weight contributions *w*_*s*_, *w*_*DB*_ and *w*_*D*_:

3 $$CD={w}_{s}\,\ast \,{p}_{S}+{w}_{DB}\,\ast \,{p}_{DB}+{w}_{D}\,\ast \,{p}_{D}$$

For computation, the score of each index was homogenized. For the Silhouette and Dunn indices, the optimum score was the maximum value that produced the greatest separation among all possible clusters. On the other hand, for the Davies-Bouldin index, the optimum score was the minimum value that returned the most compact cluster. Each index computed its score between the maximum (*max*) and minimum (*min*) values, such that the Silhouette and Dunn indices assigned a probability of 1 at *max* and 0 at *min*, while the Davies-Bouldin index assigned a probability of 1 at global *min* and 0 at *max*, defined as follows:

4 $${p}_{S}(\lambda )=\frac{S(\lambda )-\,{\min }(S)}{{\max }(S)-\,{\min }(S)}$$

5 $${p}_{DB}(\lambda )=\frac{DB(\lambda )-\,{\max }(DB)}{{\min }(DB)-\,{\max }(DB)}$$

6 $${p}_{D}(\lambda )=\frac{D(\lambda )-\,{\min }(D)}{{\max }(D)-\,{\min }(D)}$$

The expressions above enabled us to calculate the probability values for each combination (λ, from 1 to 28). The *min* and *max* values were the global extremes of each internal validation index among all the combinations (distance vs. metric). Thus, when the *CD*-index is close to 1 (or 100%), the three indices interact to produce the maximum cohesion-dispersion of the clustering (see Fig. 5). We consider that the *CD*-index is a proper validity measure for quantifying the success of the spike-sorting algorithm.

#### CE-index: clustering error measure

To validate the classification we implemented the clustering error index (*CE*-index), which measures the misclassification of the clustering. The *CE*-index has been defined as the root-mean-square difference between expected and observed values of the mean correlation coefficients:

7 $$CE=\sqrt{\frac{{\sum }_{i=1}^{n}\,{({\bar{R}}_{i}-{\rm{\Delta }})}^{2}+{\sum }_{k=1}^{m}\,{\bar{R}}_{k}^{2}}{{\sum }_{i=1}^{n}\,{\bar{R}}_{i}+{\sum }_{k=1}^{m}\,{\bar{R}}_{k}}}$$

In the expression above, ${\bar{R}}_{i}$ (with $i=1,\ldots ,n$) is the mean correlation coefficient for the relationships between the template corresponding to the *i*-cluster and all its spike events (i.e., the template vs. spikes mutual-correlations), ${\bar{R}}_{k}$ (with $k=1,\ldots ,m$) is the mean correlation coefficient for the relationships between the template corresponding to the *i*-cluster and all the spike events of another *j*-cluster (i.e., the template vs. spikes mixed-correlations, when *i ≠ j*). In this way, comparisons of all spike events with all available templates were carried out. The positive integers *n* and *m* = *n*(*n* – 1) are the number of diagonal and nondiagonal elements of the observed classification matrix, respectively. Also, Δ is the value in the diagonal of the expected classification matrix and represents the optimal mean correlation-coefficient (from the template vs. spikes mutual-correlations) to achieve the better performance of the sorting procedure (see Supplementary Appendix S5 for details). A lower *CE*-index indicates fewer misclassified and unclassified events (see Fig. 6), and therefore a better spike-sorting performance. Also, note that in accordance with Eq. (7), the value of the *CE*-index does not depend on the number of features used in the spike-sorting algorithm. The clustering error (*CE*-index) reported in Table 6 of the main text for the experimental data (rmPFC extracellular recordings) was calculated according to Eq. (7).

In order to compare our approach with other methods^24,40^ of spike sorting, we also estimated the observed and expected classification matrices, as well as, the *CE*-index and the customized Error Index (*EI*) based in the number of well-classified (${w}_{S}=\sum _{i=1}^{n}\,{N}_{{R}_{i}\in {{\rm{\Omega }}}_{i}}$), misclassified (${m}_{S}=\sum _{k=1}^{m}\,{N}_{{R}_{k}\in {{\rm{\Omega }}}_{k}}$) and unclassified [*u*_*S*_ = *N*_*S*_ − (*w*_*S*_ + *m*_*S*_)] spike events. Here, *N*_*S*_ is the total number of spike events; ${d}_{i}={N}_{{R}_{i}\in {{\rm{\Omega }}}_{i}}$ is the number of spikes for which the mutual-correlations between them and the template of their own cluster return correlation coefficients that belong to the set Ω_*i*_ [where Ω_*i*_ is the set that represents the $({\bar{R}}_{i}\times 100) \%$ of the highest correlation coefficients]; and ${r}_{k}={N}_{{R}_{k}\in {{\rm{\Omega }}}_{k}}$ is the number of spikes for which the mixed-correlations between them and the templates of other clusters return correlation coefficients that belong to the set Ω_*k*_ [where Ω_*k*_ is the set that represents the $({\bar{R}}_{k}\times 100) \%$ of the highest correlation coefficients]. Therefore, the *CE*-index was also defined as a function of *δ*_*i*_, *d*_*i*_ and *r*_*k*_, or of the customized Error Index (*EI*) reported by other authors^24^:

8 $$CE=\sqrt{\frac{\sum {({d}_{i}-{\delta }_{i})}^{2}+\sum {r}_{k}^{2}}{\sum {d}_{i}+\sum {r}_{k}}}=\sqrt{\frac{E{I}^{2}}{\sum {d}_{i}+\sum {r}_{k}}}$$

In Eq. (8), *δ*_*i*_ is the total number of spikes that we know belong to the *i*-cluster, while *d*_*i*_ and *r*_*k*_ are the diagonal and nondiagonal elements, respectively, of the observed classification matrix (see Supplementary Appendix S5 for details).

Finally, the customized Error Index (corresponding to the simulated data with three spike templates (*T*1, *T*2 and *T*3) and an important noise component) reported in Table 4 (for *K*-TOPS clustering), Table 5 (for the comparison among the different methods) and in Supplementary Table S1 (for *K*-means, first step alone) was calculated according to Eq. (9).

9 $${\rm{Error}}\,{\rm{Index}}\,(EI)=CE\sqrt{{w}_{S}+{m}_{S}}=CE\sqrt{{N}_{S}-{u}_{S}}$$

### Statistical analysis

Computed results were processed for statistical analysis using the Statistics MATLAB Toolbox and SigmaPlot 11.0 package (Sigma Plot, San Jose, CA, USA). As statistical inference procedures, both ANOVA (estimate of within-group and between-group variance, on the basis of one dependent measure) and MANOVA (estimate of variance in multiple dependent parameters across groups) were used to assess the statistical significance of differences between groups. When the assumptions of normality (Shapiro-Wilk, or Kolmogorov-Smirnov tests) and equal variance of the errors (Levene Median test) were satisfied, the corresponding statistical significance test (ANOVA *F*-test: *F*_[(*m*−1), (*m*−1)x(*n*−1), (*l*−*m*)]_ statistics, with resulting *P*-value < *p* at the predetermined significance level *p* < 0.05), was performed, with sessions as repeated measures (RM), coupled with contrast analysis when appropriate^91,92^. The orders *m* (number of groups), *n* (number of rabbits), and *l* (number of multivariate observations) were reported accompanying the *F*-statistic values^34^. When the normality assumption was not verified, the significance (*P-*value) of the *Chi-square* statistic was calculated using the ranks of the data rather than their numeric values. Thus, an ANOVA test on ranks (Kruskal-Wallis ANOVA, without RM; or Friedman RM ANOVA) was used to assess the statistical significance of differences among groups. In all the cases, the pairwise multiple-comparison analyses (Holm-Sidak, or Tukey, or Student-Newman-Keuls tests, in that order of priority) were implemented. In general, for all the statistical multivariate tests, the significance level (*P*-value) was indicated. It is common to declare a result significant if the *P*-value is less than 0.05 (*), 0.01 (**), or 0.001 (***).

Wilk’s lambda criterion and its transformation to the χ^2^ distribution used in MATLAB were used to extract significant differences from MANOVA results (cluster analysis for cells–classes–spikes classification) during the spike-sorting problem in phase space. Unless otherwise indicated, data are represented by the mean ± standard error of mean (SEM).

### Spike Sorting System/Software Overview

The proposed SS-SPDF method of feature extraction and the *K*-TOPS clustering algorithm, have been integrated in a single spike-sorting system called *VISSOR* (*Viability of Integrated Spike Sorting of Real Recordings*). *VISSOR* was developed on the MATLAB (The MathWorks, Natick, MA, USA; version 7.12.0; R2011a) platform, for detecting, identifying, and classifying neural spike events distributed across the extracellular recordings, and finally for sorting these spike events according to their derivative-based features (Table 3). Nevertheless, we would like to point out that in this work we put special emphasis on the feature extraction method (SS-SPDF) and on its clustering algorithm (*K*-TOPS) but not on the software system *VISSOR*. A ready-to-use version of the code of *VISSOR* system, now under registration, will be available upon request (E-mail: rsancam@upo.es) at http://divisiondeneurociencias.es/vissor.

## Electronic supplementary material

Supplementary Material
