# Supplementary material for: Spike sorting based on shape, phase, and distribution features, and K-TOPS clustering with validity and error indices
Source: Sci Rep. 2018 Dec 12;8:17796. doi: 10.1038/s41598-018-35491-4 (PMC6290782; doi:10.1038/s41598-018-35491-4)
Supplement: Supplementary file 1 — Supplementary Material [file 41598_2018_35491_MOESM1_ESM.pdf]

Scientific Reports

Supplementary Material

Spike sorting based on shape, phase, and distribution features, and *K*-TOPS clustering with validity and error indices

Carmen Rocío Caro-Martín, José M. Delgado-García, Agnès Gruart, and Raudel Sánchez-Campusano*

Division of Neurosciences, Pablo de Olavide University, Seville-41013, Spain

APPENDIX S1: *K*-means Clustering Overview

In the process of spike event classification, the independent feature vectors obtained after the spike feature extraction were comprised and compared to obtain the best clustering and the optimal number of clusters. The well-known *K*-means clustering method[1,2] was implemented, as its name indicates, using the means of each group of feature vectors to detect the similarities among them and the formed centers by clusters of similar feature vectors. In this paper, the *K*-means clustering method was applied as an independent step (see the resulting classification matrices and the Error Indices in Table S1) or in combination with other essential steps during spike sorting process [*K*-means + Template Optimization in the Phase Space (TOPS clustering algorithm), see Table 4 in the main text] mainly for sorting the single-unit spikes, and for both simulated data (Table S1, and Tables 4 and 5 of the main text) and experimental recordings (Table 6 of the main text).

K-means clustering method had the purpose of maximizing the similarity of feature vectors within each clustering and of minimizing the similarity among feature vectors of different clustering[3]. This method was previously described by Tou and González (1974)[4] in the following steps:

1. *Establish the initial conditions of the classification centers*. By default, values of the feature vectors array were chosen as initial values of classification centers. In additions, the number of chosen centers was equal to the number of final clustering obtained from the classification.
2. *Apply distance and metric measurement criteria among the different feature vectors and among the centroids*. Four different measures of distance (sqEuclidean, Cityblock, Cosine and Correlation; see Table S2 for details) among clusters and seven clustering metrics (sqEuclidean, Euclidean, Cityblock, Cosine, Correlation, Hamming and Jaccard; see Table S3 for details) were considered. Therefore, a total of twenty-eight (4 distances x 7 metrics) different combinations were applied in the classification (Fig. S1).
3. *Calculate the membership of the feature vectors to a cluster until convergence criteria.* Given an initial but not optimal clustering, relocated each features vector to its new nearest center, updated the clustering centers by calculating the mean of the feature vectors clustered, and repeated the relocating-and-updating process until that the convergence criteria (such as predefined number of iterations) were satisfied.
4. Finally, *K*-means clustering method returned the most pertinent *K*-value (optimal number of clusters) and the most suitable classification (optimal clustering) of the feature vectors for each distance-metric combination and for the indicated number of centroids.

In addition, three internal validation indices [Silhouette (*S*), Davies-Bouldin (*DB*) and Dunn indices (*D*), see the Appendices S2–S4 below] were used according to the diagram of the supplementary Fig. S1. In this way, supplementary Fig. S2 shows the values (in %) of the internal validation indices (Silhouette, Dunn, and Davies-Bouldin) for all the combinations (distance vs. metric) after applying *K*-TOPS clustering (*K*-means and template optimization in phase space) on neuronal spike events from the first derivative of the rmPFC filtered record (epoch of 1.5 s). For each selected distance, the resulting number of clusters according to the criterion of the extreme values (*max* and *min*) of the internal validation indices was different (sqEuclidean, $K = 5$; Cityblock, $K = 3$; Cosine, $K = 5$; Correlation, $K = 4$) and therefore suboptimal.

Moreover, for some combinations (sqEuclidean vs. Cosine; sqEuclidean vs. Correlation; Cityblock vs. sqEuclidean; Cityblock vs. Euclidean; Cityblock vs. Cityblock; Cosine vs. Hamming; Cosine vs. Jaccard; Correlation vs. Euclidean; and Correlation vs. Cityblock) the criterion was not met (indicated as a failure in the corresponding combination, see Fig. S2b-e).

However, the goal of the spike classification was to find a single optimal value for the number of clusters and the best clustering.

For this purpose, a proper validity measure (*CD*-index) was implemented in this work to obtain the optimal number of clusters among all the distance-metric combinations. The main goal was to make the three internal validation indices interact among themselves to produce the maximum cohesion-dispersion of the clustering across all the distance-metric combinations (see Methods in the main text). In combination with the validity index above (*CD*-index) for determining the optimal number of clusters, a customized clustering error index (*CE*-index) was applied for determining the optimal clustering (see Methods in the main text and the Supplementary Appendix S5 for details).

APPENDIX S2: Silhouette Index

The Silhouette (S) index value for each point i is a measure of the similarity of a point to points in its own cluster compared with points in other clusters[5]. For a given grouping $\mathcal{A}_j\ j \in [1, \dots, c]$; c = number of classes, the method assigns each object $\mathcal{A}_j$ a quality measure $\mathcal{S}_i\ (i = 1, \dots, m)$; m =number of objects of $\mathcal{A}_j$. The Silhouette index value is defined as:

$$\mathcal{S}(i) = \frac{b(i) - a(i)}{\max\{a(i), b(i)\}}$$

In the above expression, $a(i)$ is the average distance between the *i-th* object and all the objects included in $\mathcal{A}_j$, while $b(i)$ is the minimum distance between the *i-th* object and all objects classified in $\mathcal{A}_k\ k \in [1, \dots, c]$, $k \neq j$.

The index value will be in the range $-1 \leq \mathcal{S}(i) \leq 1$, and any object being poorly grouped will have an index value $-1 \leq \mathcal{S}(i) \leq 0$. The index for a group $\mathcal{A}_j$ is defined as:

$$\mathbb{S}_j = \frac{1}{m}\sum_{i=1}^{m} \mathcal{S}(i)$$

Here m is the number of objects within $\mathcal{A}_j$ and c is the total number of classes. Therefore, the average value of the Silhouette index indicating whether the classification made is optimal, adequate, and well separated so as to provide the optimal number of classes is as follows:

$$S = \frac{1}{c}\sum_{j=1}^{c} \mathbb{S}_j$$

APPENDIX S3: Davies-Bouldin Index

This index measures the average similarity of groups of objects (classes) within a classification. A low value of the Davies-Bouldin (*DB*) index indicates that a class is grouped by similar objects, is compact, and has the centers of each class far from each other[6]. The average value of the index for different classes determines the final value of *DB*. This index is defined with the following formulation:

$$DB = \frac{1}{c}\sum_{j=1}^{c} max_{i\neq j}\left\{\frac{\Lambda(\mathcal{A}_j) + \Lambda(\mathcal{A}_i)}{\Lambda(\mathcal{A}_j, \mathcal{A}_i)}\right\}$$

In the above expression, $\mathcal{A}_j$ and $\mathcal{A}_i$ represent the class *j* and *i,* respectively; $\mathcal{A}_i$ represents the class *i;* $\Lambda(\mathcal{A}_j, \mathcal{A}_i)$ the distance between classes $\mathcal{A}_j$ and $\mathcal{A}_i$; the term $\Lambda(\mathcal{A}_k)$ represents the distances within the class $\mathcal{A}_k$; and finally *c* is the number of similar classes of objects within the classification $\mathrm{C}\left\{\cup\mathcal{A}_j \;\; j \in [1, \dots, c]\right\}$.

APPENDIX S4: Dunn Index

This index maximizes the distances within each class of similar objects while minimizing the distances between the remaining classes of the classification – i.e., it identifies compact, well separated classes[7]. Therefore, high values of Dunn's index indicate the presence of very compact classes and separate objects within a classification. The number of classes that maximizes the *D*-index is taken as number of optimal classes. For any classification $\mathrm{C}\left\{\cup\mathcal{A}_j \;\; j \in [1, \dots, c]\right\}$, the *D*-index is defined as:

$$D(\mathrm{C}) = min_{1\leq j\leq c}\left\{min_{1\leq i\leq c}\left\{\frac{\Lambda(\mathcal{A}_j, \mathcal{A}_i)}{max_{1\leq k\leq c}\{\Lambda(\mathcal{A}_k)\}}\right\}\right\}$$

Here, $\Lambda(\mathcal{A}_j, \mathcal{A}_i)$ denotes the distance between classes $\mathcal{A}_j$ and $\mathcal{A}_i$; $\Lambda(\mathcal{A}_k)$ represents the distances within the class $\mathcal{A}_k$; and *c* is the number of similar classes of objects within the classification $\mathrm{C}\left\{\cup\mathcal{A}_j \;\; j \in [1, \dots, c]\right\}$.

APPENDIX S5: *CE*-index

The *CE*-index quantifies the similitude between the observed classification matrix and the expected classification matrix. This index is an adaptation of the error index proposed by Letelier

and Weber (2000)[8]. In the present work, the observed classification matrix, based on the mean correlation coefficients (template vs. spikes), was defined as:

$$\begin{array}{c} \\ C1 \\ C2 \\ C3 \\ \vdots \\ Cn \end{array} \begin{array}{c} \begin{array}{ccccc} T1 & T2 & T3 & \cdots & Tn \end{array} \\ \begin{pmatrix} \bar{R}_{11} & \bar{R}_{12} & \bar{R}_{13} & \cdots & \bar{R}_{1n} \\ \bar{R}_{21} & \bar{R}_{22} & \bar{R}_{23} & \cdots & \bar{R}_{2n} \\ \bar{R}_{31} & \bar{R}_{32} & \bar{R}_{33} & \cdots & \bar{R}_{3n} \\ \vdots & \vdots & \vdots & \ddots & \vdots \\ \bar{R}_{n1} & \bar{R}_{n2} & \bar{R}_{n3} & \cdots & \bar{R}_{nn} \end{pmatrix} \end{array}$$

In the case of the existence of n clusters resulting from the classification, Ti represents the spikes belonging to the i-template, while the clusters Ci represent the groups distinguished by the automatic clustering algorithm. Here, $\bar{R}_{ii}$ are the mean correlation coefficients for the relationships between the template corresponding to the i-cluster and all its spike events (i.e., the template vs. spikes mutual-correlations), $\bar{R}_{ij}$ are the mean correlation coefficients for the relationships between the template corresponding to the i-cluster and all the spike events of another j-cluster (i.e., the template vs. spikes mixed-correlations, when $i \neq j$). In this way, comparisons of all spike events with all available templates were carry out.

A perfect performance of the sorting procedure, at $\Delta = \bar{R}_{ii} = 1$ (i.e., the strongest mutual-correlations possible) with $i = 1, \ldots, n$, should produce the following expected classification matrix:

$$\begin{array}{c} \\ C1 \\ C2 \\ C3 \\ \vdots \\ Cn \end{array} \begin{array}{c} \begin{array}{ccccc} T1 & T2 & T3 & \cdots & Tn \end{array} \\ \begin{pmatrix} \Delta & 0 & 0 & \cdots & 0 \\ 0 & \Delta & 0 & \cdots & 0 \\ 0 & 0 & \Delta & \cdots & 0 \\ \vdots & \vdots & \vdots & \ddots & \vdots \\ 0 & 0 & 0 & \cdots & \Delta \end{pmatrix} \end{array}$$

However, in practice, during automatic clustering algorithm, the Δ-value could be adjusted explicitly to achieve better performance according to $\Delta = (\max(\bar{R}_{ii}) + n)/(n + 1)$, because the perfect performance ($\Delta = 1$) is a very strong theoretical requirement. Note that, $\mathcal{R} = \bar{R}_{ij} = 0$ (i.e., the weakest mixed-correlations possible) and therefore, there is no linear relationship between the clusters. Finally, the *CE*-index was defined as the root-mean-square difference between observed and expected values of the mean correlation coefficients:

$$CE = \sqrt{\frac{\sum_{i=1}^{n}(\bar{R}_i - \Delta)^2 + \sum_{k=1}^{m}\bar{R}_k^2}{\sum_{i=1}^{n}\bar{R}_i + \sum_{k=1}^{m}\bar{R}_k}}$$

In equation above, $i = 1, \dots, n$ and $k = 1, \dots, m$, where the positive integers n and $m = n(n-1)$ are the number of diagonal and nondiagonal elements, respectively, of the observed classification matrix. The clustering error (CE-index) reported in Table 6 of the main text for the experimental data (rmPFC extracellular recordings) was calculated according to the equation above.

Furthermore, we quantified well-classified ($w_S = \sum_{i=1}^{n} N_{R_i \in \Omega_i}$), misclassified ($m_S = \sum_{k=1}^{m} N_{R_k \in \Omega_k}$) and unclassified [$u_S = N_S - (w_S + m_S)$] profiles. Here, N_S is the total number of spike events; $d_i = N_{R_i \in \Omega_i}$ is the number of spikes for which the mutual-correlations between them and the template of their own cluster return correlation coefficients that belong to the set Ω_i [where Ω_i is the set that represents the $(\bar{R}_i \times 100)\%$ of the highest correlation coefficients]; and $r_k = N_{R_k \in \Omega_k}$ is the number of spikes for which the mixed-correlations between them and the templates of other clusters return correlation coefficients that belong to the set Ω_k [where Ω_k is the set that represents the $(\bar{R}_k \times 100)\%$ of the highest correlation coefficients].

In order to compare our approach with other methods[8,9] of spike sorting, we also estimated the observed and expected classification matrices, as well as, the *CE*-index and the customized Error Index (*EI*) based in the number of well-classified ($w_S = \sum d_i$), misclassified ($m_S = \sum r_k$) and unclassified [$u_S = N_S - (\sum d_i + \sum r_k)$] spike events, where d_i and r_k are the diagonal and nondiagonal elements, respectively, of the observed classification matrix. Here, δ_i is the total number of spikes that we know belong to the *i*-cluster.

$$CE = \sqrt{\frac{\sum_{i=1}^{n}(N_{R_i \in \Omega_i} - \delta_i)^2 + \sum_{k=1}^{m} N_{R_k \in \Omega_k}^2}{\sum_{i=1}^{n} N_{R_i \in \Omega_i} + \sum_{k=1}^{m} N_{R_k \in \Omega_k}}} = \sqrt{\frac{\sum (d_i - \delta_i)^2 + \sum r_k^2}{\sum d_i + \sum r_k}} = \sqrt{\frac{EI^2}{\sum d_i + \sum r_k}}$$

$$\text{Error Index } (EI) = CE\sqrt{w_S + m_S} = CE\sqrt{N_S - u_S}$$

Finally, the customized Error Index (corresponding to the simulated data with three spike templates (*T*1, *T*2 and *T*3) and an important noise component) reported in Table S1 (for *K*-means, first step alone), in Table 4 (for *K*-TOPS clustering algorithm) and in Table 5 (for the comparison among the different methods) of the main text, was calculated according to the equation above.

Supplementary References

1. MacQueen, J. Some methods for classification and analysis of multivariate observations *In Proc. 5th Berkeley Sym. Math. Stat. Prob.* 281–297 (University of California Press, Oakland, 1967).
2. Jim, X. & Han, J. K-means clustering In *Encyclopedia of Machine Learning* (Eds. Sammut C and Webb G J) 563–564 (Springer U.S., 2010).
3. Lewicki, M. S. A review of methods for spike sorting: the detection and classification of neural action potentials. *Network* **9**, 53–78 (1998).
4. Tou, J. T., & González, R. C. *Pattern Recognition Principles* (Massachusetts: Addixon-Wesley, 1974).
5. Rousseeuw, P. J. Silhouettes: a graphical aid to the interpretation and validation of cluster analysis. *J. Comput. Appl. Math.* **20**, 53–65 (1987).
6. Davies, D. L., & Bouldin, D. W. A cluster separation measure. *IEEE Trans. Pattern. Anal. Mach. Intell.* **1**, 224–227 (1979).
7. Dunn, J. C. Well separated clusters and optimal fuzzy partitions. *J. Cybern.* **4**, 95–104 (1970).
8. Letelier, J. C., & Weber, P. P. Spike sorting based on discrete wavelet transform coefficients. *J. Neurosci. Methods* **101**, 93–106 (2000).
9. Aksenova, T. I., Chibirova, O. K., Dryga, O. A., Tetko, I. V., Benabid, A. L., & Villa, A. E. P. An unsupervised automatic method for sorting neuronal spike waveforms in awake and freely moving animals. *Methods* **30**, 178–187 (2003).

Supplementary Tables

Table S1. Observed classification matrices resulting from unsupervised *K*-means clustering algorithm (first step alone) for sorting the single-unit spikes, on five simulated datasets (from D_1 to D_5). The first number in unclassified, misclassified or well-classified columns is the number of spike events. The number in parentheses represents the number of overlapping waveforms for each category. In the last row, the corresponding percentage values (%) are indicated. In the last column, the resulting Error Index [see Eq. (9) in the main text] for each simulated dataset and the average value of them are reported.

Dataset	Classification Matrix	*Unclassified	*Misclassified	*Well-classified	Error Index
**D_1**	$\begin{matrix} & T1 & T2 & T3 \end{matrix}$ $\begin{matrix} C1 \\ C2 \\ C3 \end{matrix}\begin{pmatrix} 2700 & 3 & 1 \\ 4 & 2683 & 1 \\ 0 & 0 & 2698 \end{pmatrix}$	91 (81)	9 (0)	8081 (0)	58.677
**D_2**	$\begin{matrix} & T1 & T2 & T3 \end{matrix}$ $\begin{matrix} C1 \\ C2 \\ C3 \end{matrix}\begin{pmatrix} 2696 & 4 & 1 \\ 4 & 2692 & 1 \\ 0 & 0 & 2699 \end{pmatrix}$	84 (81)	10 (0)	8087 (0)	57.376
**D_3**	$\begin{matrix} & T1 & T2 & T3 \end{matrix}$ $\begin{matrix} C1 \\ C2 \\ C3 \end{matrix}\begin{pmatrix} 2699 & 5 & 1 \\ 4 & 2689 & 1 \\ 0 & 0 & 2697 \end{pmatrix}$	85 (81)	11 (0)	8155 (0)	57.732
**D_4**	$\begin{matrix} & T1 & T2 & T3 \end{matrix}$ $\begin{matrix} C1 \\ C2 \\ C3 \end{matrix}\begin{pmatrix} 2697 & 4 & 1 \\ 4 & 2691 & 1 \\ 0 & 0 & 2695 \end{pmatrix}$	88 (81)	10 (0)	8145 (0)	59.666
**D_5**	$\begin{matrix} & T1 & T2 & T3 \end{matrix}$ $\begin{matrix} C1 \\ C2 \\ C3 \end{matrix}\begin{pmatrix} 2698 & 9 & 1 \\ 4 & 2690 & 1 \\ 0 & 0 & 2696 \end{pmatrix}$	82 (81)	15 (0)	8152 (0)	59.195
**Mean**	$\begin{matrix} & T1 & T2 & T3 \end{matrix}$ $\begin{matrix} C1 \\ C2 \\ C3 \end{matrix}\begin{pmatrix} 2698 & 5 & 1 \\ 4 & 2689 & 1 \\ 0 & 0 & 2697 \end{pmatrix}$	86 (81)<br>Percentage (%)<br>1.05% (100%)	11 (0)<br>Percentage (%)<br>0.13% (0.00%)	8084 (0)<br>Percentage (%)<br>98.81% (0.00%)	58.529<br>SEM<br>0.431

*Here are indicated, well-classified ($w_S = \sum d_i$), misclassified ($m_S = \sum r_k$) and unclassified [$u_S = N_S - (\sum d_i + \sum r_k)$] spike events. Also, N_S is the total number of spike events (8181, among which 8100 are single-unit spikes and 81 are overlapping waveforms), while d_i and r_k are the diagonal and nondiagonal elements of the observed classification matrix, respectively. Abbreviations: SEM, standard error of the mean.

Table S2. Summary of the available distance measures by *K*-means. *K*-means computes centroid clusters differently for the different supported distance measures. In the formulae, x is an observation (that is, a row of the matrix X) and c is a centroid (a row vector).

Distance	Description	Algebraic definition
**SqEuclidean**	Squared Euclidean distance (default). Each centroid is the mean of the points in that cluster.	$d(x,c) = (x-c)(x-c)'$
**Cityblock**	Sum of absolute differences. Each centroid is the component-wise median of the points in that cluster.	$d(x,c) = \sum_{j=1}^{p} \lvert x_j - c_j \rvert$
**Cosine**	One minus the cosine of the included angle between points (treated as vectors). Each centroid is the mean of the points in that cluster, after normalizing those points to unit Euclidean length.	$d(x,c) = 1 - \frac{xc'}{\sqrt{(xx')(cc')}}$
**Correlation**	One minus the sample correlation between points (treated as sequences of values). Each centroid is the component-wise mean of the points in that cluster, after centering and normalizing those points to zero mean and unit standard deviation.	$d(x,c)$ $= 1 - \frac{(x-\vec{\bar{x}})(c-\vec{\bar{c}})'}{\sqrt{(x-\vec{\bar{x}})(x-\vec{\bar{x}})'}\sqrt{(c-\vec{\bar{c}})(c-\vec{\bar{c}})'}}$ where $\vec{\bar{x}} = \frac{1}{p}(\sum_{j=1}^{p} x_j)\overrightarrow{1_p}$ $\vec{\bar{c}} = \frac{1}{p}(\sum_{j=1}^{p} c_j)\overrightarrow{1_p}$ $\overrightarrow{1_p}$ es a row vector of p ones

Table S3. Summary of the available metric measures. The algorithm calculates the internal validation indices using the inter-point distance function specified as metric.

Metric	Description
**Euclidean**	Euclidean distance.
**sqEuclidean**	Squared Euclidean distance (default).
**Cityblock**	Sum of absolute differences.
**Cosine**	One minus the cosine of the included angle between points (treated as vectors).
**Correlation**	One minus the sample correlation between points (treated as sequences of values).
**Hamming**	Percentage of coordinates that differ.
**Jaccard**	Percentage of nonzero coordinates that differ.

Table S4. Statistical reports corresponding to the comparison of the mean values of the *CE*-index employing different feature vectors (FV2, FV3, FV5, FV6, and FV24; see Table 6 for description) to check the clustering performance from a session of recordings [n = 60 trials, from the rostral-medial prefrontal cortex (rmPFC)]. Here, the values of the statistics *H* (One-Way ANOVA on Ranks) and *F* (One-Way ANOVA *F*-test) are reported. The significance level (*P*-value) is indicated in each case.

**Many Groups (Central Measurement)**	**Statistical Test**	**Report**	***P*-value**
**FV2 vs. FV3 vs. FV5 vs. FV6 vs. FV24**	Kruskal-Wallis One-Way ANOVA on Ranks	$H = 62.091$ with 4 degrees of freedom	$P < 0.001$; ***
**FV2 vs. FV5 vs. FV24**	Kruskal-Wallis One-Way ANOVA on Ranks	$H = 50.365$ with 2 degrees of freedom	$P < 0.001$; ***
**FV3 vs. FV6 vs. FV24**	Kruskal-Wallis One-Way ANOVA on Ranks	$H = 7.049$ with 2 degrees of freedom	$P = 0.029$; *
**Two Groups (Pairwise comparison)**	**Statistical Test**	**Report**	***P*-value**
FV2 vs. FV24	Kruskal-Wallis One-Way ANOVA on Ranks	$H = 39.576$ with 1 degree of freedom	$P < 0.001$; ***
FV3 vs. FV24	One-Way ANOVA *F*-test	$F_{(1,12,118)} = 7.537$	$P = 0.007$; **
FV5 vs. FV24	Kruskal-Wallis One-Way ANOVA on Ranks	$H = 33.550$ with 1 degree of freedom	$P < 0.001$; ***
FV6 vs. FV24	One-Way ANOVA *F*-test	$F_{(1,12,118)} = 4.822$	$P = 0.030$; *
FV2 vs. FV3	Kruskal-Wallis One-Way ANOVA on Ranks	$H = 18.549$ with 1 degree of freedom	$P < 0.001$; ***
FV2 vs. FV5	Kruskal-Wallis One-Way ANOVA on Ranks	$H = 2.639$ with 1 degree of freedom	$P = 0.104$
FV2 vs. FV6	Kruskal-Wallis One-Way ANOVA on Ranks	$H = 22.169$ with 1 degree of freedom	$P < 0.001$; ***
FV3 vs. FV5	Kruskal-Wallis One-Way ANOVA on Ranks	$H = 11.462$ with 1 degree of freedom	$P < 0.001$; ***
FV3 vs. FV6	Kruskal-Wallis One-Way ANOVA on Ranks	$H = 0.211$ with 1 degree of freedom	$P = 0.646$
FV5 vs. FV6	Kruskal-Wallis One-Way ANOVA on Ranks	$H = 16.655$ with 1 degree of freedom	$P < 0.001$; ***

Table S5. Values of the parameter $\boldsymbol{C}$ for determining the feature vector dimensionality as a function of the electrode array density. Here the number of electrodes is expressed as powers of 2 (that is, $\boldsymbol{N_{electrodes}} = \boldsymbol{2^n}$; for example: 2^0 = 1 electrode; 2^2 = 4 electrodes = 1 tetrode; 2^5 = 32 electrodes; 2^7 = 128 electrodes; 2^{10} = 1024 electrodes; and so on until n = 19) to show the robustness of the selected criterion*, but any discrete value (not power of 2) could be selected. In summary, with $\boldsymbol{C < 0.5}$ and $\boldsymbol{C < 0.25}$ the following relations between $\boldsymbol{N_{electrodes}}$ and $\boldsymbol{N_{features}}$ can be obtained. For $\boldsymbol{C < 0.5}$ (see yellow cells): [from 2^0 to 2^4 electrodes, 24 features]; [2^5 electrodes, 18 features]; [2^6 electrodes, 14 features]; [2^7 electrodes, 13 features]; [from 2^8 to 2^{10} electrodes, 12 features]; and [from 2^{11} to 2^{19} electrodes, 11 features]. For $\boldsymbol{C < 0.25}$ (see green cells): [from 2^0 to 2^2 electrodes, 24 features]; [2^3 electrodes, 22 features]; [2^4 electrodes, 9 features]; [2^5 electrodes, 7 features]; [from 2^6 to 2^8 electrodes, 6 features]; and [from 2^9 to 2^{19} electrodes, 5 features].

$N_{features}$	$N_{electrodes}$											
	2^0	2^1	2^2	2^3	2^4	2^5	2^6	2^7	2^8	2^9	2^{10}	2^{19}
**24**	0.04	0.08	0.14	**0.25**	**0.40**	0.67	0.73	0.84	0.91	0.96	0.98	1.00
**23**	0.04	0.08	0.14	**0.25**	**0.40**	0.56	0.70	0.81	0.88	0.92	0.94	0.96
**22**	0.04	0.08	0.14	0.24	**0.39**	0.54	0.68	0.78	0.84	0.87	0.90	0.92
**21**	0.04	0.08	0.14	0.24	**0.38**	0.53	0.66	0.75	0.81	0.84	0.86	0.88
**20**	0.04	0.08	0.14	0.24	**0.37**	0.51	0.64	0.72	0.77	0.80	0.82	0.83
**19**	0.04	0.08	0.14	0.24	**0.36**	0.50	0.61	0.69	0.74	0.76	0.78	0.79
**18**	0.04	0.08	0.14	0.23	**0.35**	**0.48**	0.58	0.66	0.70	0.72	0.74	0.75
**17**	0.04	0.07	0.13	0.23	**0.34**	**0.46**	0.56	0.63	0.66	0.69	0.70	0.71
**16**	0.04	0.07	0.13	0.22	**0.33**	**0.44**	0.53	0.59	0.63	0.65	0.66	0.67
**15**	0.04	0.07	0.13	0.22	**0.32**	**0.43**	0.51	0.56	0.59	0.61	0.62	0.63
**14**	0.04	0.07	0.13	0.21	**0.31**	**0.41**	**0.48**	0.53	0.55	0.57	0.58	0.58
**13**	0.04	0.07	0.13	0.21	**0.30**	**0.39**	**0.45**	**0.49**	0.52	0.53	0.53	0.54
**12**	0.04	0.07	0.12	0.20	**0.29**	**0.36**	**0.42**	**0.46**	**0.48**	**0.49**	**0.49**	0.50
**11**	0.04	0.07	0.12	0.19	**0.27**	**0.34**	**0.39**	**0.42**	**0.44**	**0.45**	**0.45**	**0.46**
**10**	0.04	0.07	0.12	0.19	**0.26**	**0.32**	**0.36**	**0.39**	**0.40**	**0.41**	**0.41**	**0.42**
**9**	0.04	0.07	0.12	0.18	0.24	**0.29**	**0.33**	**0.35**	**0.36**	**0.37**	**0.37**	**0.38**
**8**	0.04	0.07	0.11	0.17	0.22	**0.27**	**0.30**	**0.31**	**0.32**	**0.33**	**0.33**	**0.33**
**7**	0.04	0.06	0.11	0.16	0.20	0.24	**0.26**	**0.28**	**0.28**	**0.29**	**0.29**	**0.29**
**6**	0.04	0.06	0.10	0.14	0.18	0.21	0.23	0.24	0.24	**0.25**	**0.25**	**0.25**
**5**	0.03	0.06	0.09	0.13	0.16	0.18	0.19	0.20	0.20	0.21	0.21	0.21
**4**	0.03	0.06	0.08	0.11	0.13	0.15	0.16	0.16	0.16	0.17	0.17	0.17
**3**	0.03	0.05	0.07	0.09	0.11	0.11	0.12	0.12	0.12	0.12	0.12	0.12

*Criterion: $\left(\sum_{i=1}^{N_{features}} W(f_i)/N_{features}\right) \times \left(N_{electrodes}/\left(N_{electrodes} + N_{features}\right)\right) < C$; where $\sum_{i=1}^{N_{features}} W(f_i)$ is the sum of weights off all features, $N_{features}$ and $N_{electrodes}$ are the numbers of features and electrodes, respectively.

Table S6. Execution times across the training sessions (C1 – C10) employing feature vectors with different features and dimensions (FV2, FV3, FV5, FV6, and FV24; see Table 6 for description). The values of the execution times are reported by the Mean ± SEM (standard error of the mean). Here, the values of the statistics *H* (One-Way ANOVA on Ranks) and *F* (One-Way ANOVA *F*-test) are reported. The significance level (*P*-value) is indicated in each case.

Session	FV	Total execution time (in minutes) for each session (60 trials) of recordings	
		**Mean ± SEM**	**Statistical report**
**C1**	FV2	53.097 ± 0.540	Kruskal-Wallis One Way ANOVA on Ranks.
	FV3	53.827 ± 0.727	*H* = 3.922 with 4 degrees of freedom (i.e., among five groups).
	FV5	54.232 ± 1.084	The differences in the median values among the treatment groups are not great
	FV6	53.439 ± 0.634	enough to exclude the possibility that the difference is due to random sampling
	FV24	54.849 ± 0.650	variability; there is not a statistically significant difference (*P* = 0.417).
**C2**	FV2	54.126 ± 0.860	One Way ANOVA *F*-test.
	FV3	54.984 ± 1.232	*F* = 0.334 with 4 degrees of freedom (five groups).
	FV5	53.660 ± 1.043	The differences in the mean values among the treatment groups are not great
	FV6	54.919 ± 0.669	enough to exclude the possibility that the difference is due to random sampling
	FV24	54.370 ± 0.908	variability; there is not a statistically significant difference (*P* = 0.854).
**C3**	FV2	50.411 ± 0.948	Kruskal-Wallis One Way ANOVA on Ranks.
	FV3	51.229 ± 1.076	*H* = 2.830 with 4 degrees of freedom (five groups).
	FV5	51.064 ± 0.857	The differences in the median values among the treatment groups are not great
	FV6	51.852 ± 0.615	enough to exclude the possibility that the difference is due to random sampling
	FV24	50.599 ± 0.471	variability; there is not a statistically significant difference (*P* = 0.587).
**C4**	FV2	53.362 ± 1.063	One Way ANOVA *F*-test.
	FV3	53.408 ± 1.209	*F* = 0.178 with 4 degrees of freedom (five groups).
	FV5	53.658 ± 1.050	The differences in the mean values among the treatment groups are not great
	FV6	52.642 ± 0.667	enough to exclude the possibility that the difference is due to random sampling
	FV24	52.989 ± 0.549	variability; there is not a statistically significant difference (*P* = 0.948).
**C5**	FV2	51.085 ± 0.495	Kruskal-Wallis One Way ANOVA on Ranks.
	FV3	51.936 ± 0.988	*H* = 4.170 with 4 degrees of freedom (five groups).
	FV5	52.569 ± 0.934	The differences in the median values among the treatment groups are not great
	FV6	52.207 ± 0.423	enough to exclude the possibility that the difference is due to random sampling
	FV24	51.236 ± 0.344	variability; there is not a statistically significant difference (*P* = 0.383).
**C6**	FV2	51.132 ± 0.421	One Way ANOVA *F*-test.
	FV3	52.100 ± 0.843	*F* = 1.509 with 4 degrees of freedom (five groups).
	FV5	52.258 ± 0.367	The differences in the mean values among the treatment groups are not great
	FV6	52.515 ± 0.405	enough to exclude the possibility that the difference is due to random sampling
	FV24	51.196 ± 0.390	variability; there is not a statistically significant difference (*P* = 0.216).
**C7**	FV2	53.001 ± 1.148	Kruskal-Wallis One Way ANOVA on Ranks.
	FV3	54.162 ± 0.979	*H* = 2.831 with 4 degrees of freedom (five groups).
	FV5	54.915 ± 1.109	The differences in the median values among the treatment groups are not great
	FV6	53.775 ± 0.824	enough to exclude the possibility that the difference is due to random sampling
	FV24	53.098 ± 0.872	variability; there is not a statistically significant difference (*P* = 0.588).
**C8**	FV2	39.407 ± 0.737	One Way ANOVA *F*-test.
	FV3	39.566 ± 0.647	*F* = 0.507 with 4 degrees of freedom (five groups).
	FV5	40.099 ± 0.374	The differences in the mean values among the treatment groups are not great
	FV6	40.295 ± 0.459	enough to exclude the possibility that the difference is due to random sampling
	FV24	40.321 ± 0.679	variability; there is not a statistically significant difference (*P* = 0.731).
**C9**	FV2	59.193 ± 0.963	Kruskal-Wallis One Way ANOVA on Ranks.
	FV3	59.606 ± 1.383	*H* = 0.686 with 4 degrees of freedom (five groups).
	FV5	59.882 ± 1.262	The differences in the median values among the treatment groups are not great
	FV6	60.018 ± 0.599	enough to exclude the possibility that the difference is due to random sampling
	FV24	59.023 ± 1.125	variability; there is not a statistically significant difference (*P* = 0.953).
**C10**	FV2	56.242 ± 0.973	One-way ANOVA *F*-test.
	FV3	55.053 ± 0.889	*F* = 0.456 with 4 degrees of freedom (five groups).
	FV5	55.153 ± 0.942	The differences in the median values among the treatment groups are not great
	FV6	56.120 ± 0.584	enough to exclude the possibility that the difference is due to random sampling
	FV24	55.136 ± 0.905	variability; there is not a statistically significant difference (*P* = 0.768).

Supplementary Figures

Figure S1

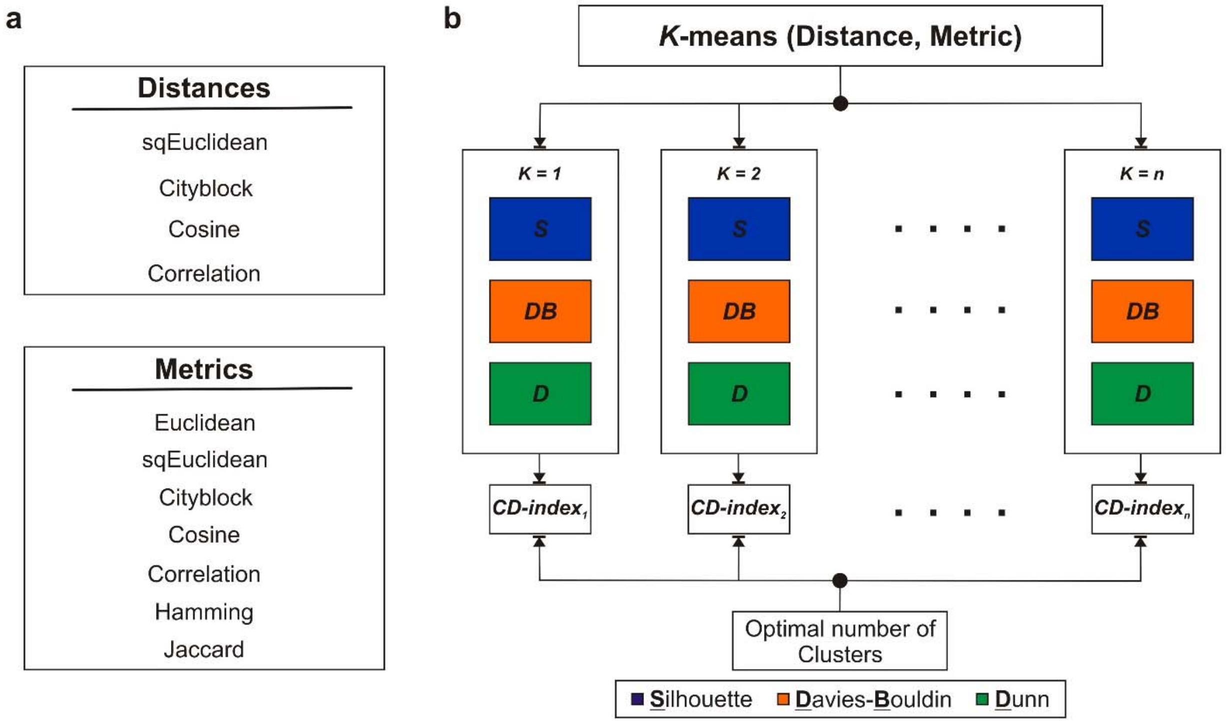

Figure S1. Unsupervised *K*-means method and internal validation indices to obtain the optimal number of clusters. (A) *K*-means method uses four different measures of distance (sqEeuclidean, Cityblock, Cosine and Correlation, in Table S1) and seven clustering metrics (sqEuclidean, Euclidean, Cityblock, Cosine, Correlation, Hamming and Jaccard, in Table S2) for all possible values of *K*. (B) In this diagram *K*-means method (for a selected distance-metric combination) uses the internal validation indices: Silhouette (S), Davies-Bouldin (DB) and Dunn (D). Also, a general index for measuring the cohesion and dispersion of the clustering (called *CD*-index) for each classification is calculated. The higher value of the *CD*-index allows determining the optimal number of clusters. Note that, the maximum number of clusters n should not exceed the value $\sqrt{s}$ where *s* is the number of detected spikes in the neural recording.

Figure S2

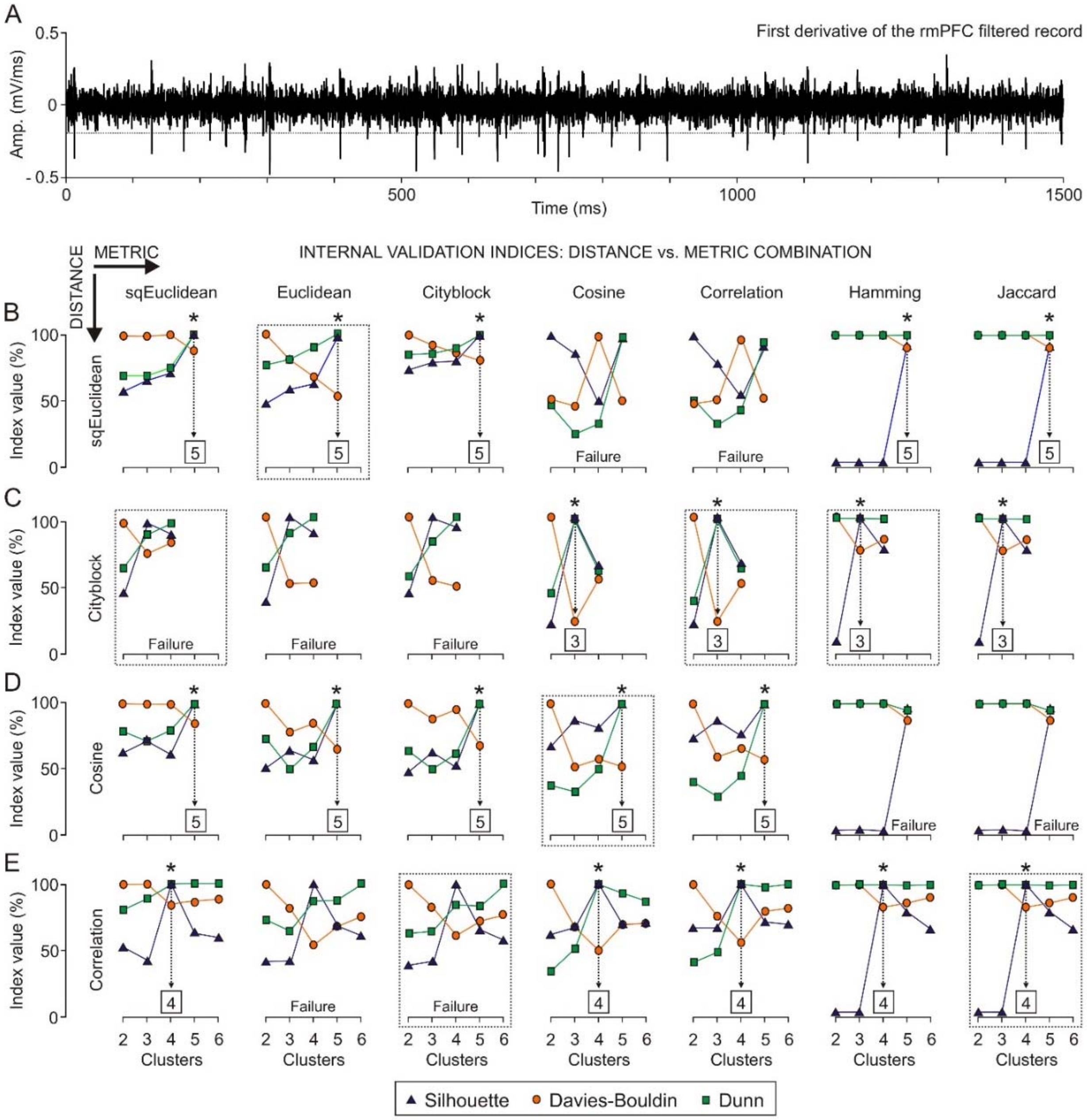

Figure S2. Automatic *K*-means clustering and the internal validation indices. (**a**) An example of the first derivative of rmPFC filtered record representing a single trial of the leaning process. The horizontal dotted line indicates the amplitude threshold. (**b-e**) Combination of all possible distances (sqEuclidean, Cityblock, Cosine, and Correlation) and metrics (sqEuclidean, Euclidean, Cityblock, Cosine, Correlation, Hamming, and Jaccard) used by the *K*-means clustering algorithm for the 24D feature vectors (Table 3). Each panel indicates the relationship between the value (in %) of the internal validation index (Silhouette, blue triangle; Davies-Bouldin, orange circle; or Dunn, green square) and the number of clusters obtained by the unsupervised method. Combinations (distance vs. metric) for which the Silhouette and Dunn indices reached their maximum values while the Davies-Bouldin index reached its minimum value are marked with an asterisk (*). In each of these cases, the suboptimal number of clusters is indicated (number inside the square). Gray dotted squares enclose some examples (one for each metric) considering different combinations (distance vs metric) for their comparative analysis in Fig. 5 of the main text, employing the cohesion-dispersion index (*CD*-index).

Figure S3

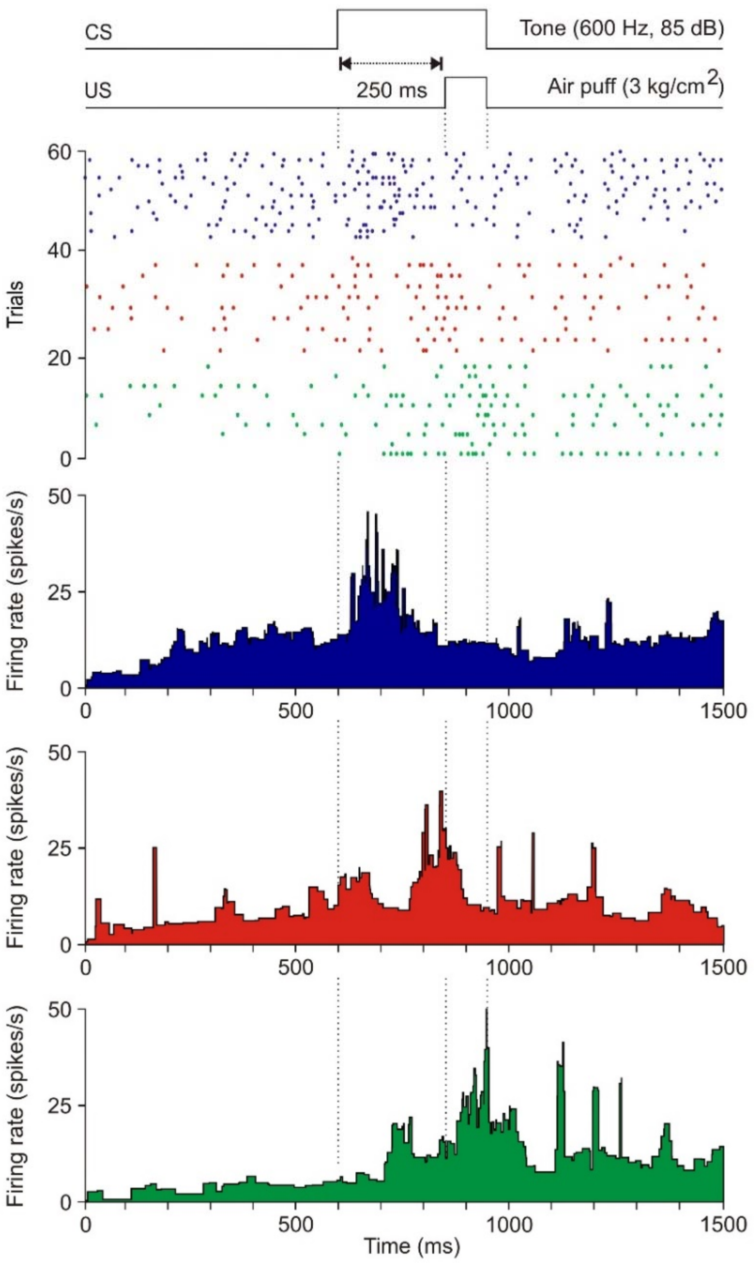

Figure S3. Results after a full implementation of the SS-SPDF method/algorithm for a representative session (60 CS–US trials) of rmPFC recordings. Firing activities of recorded neurons from the rostro-medial prefrontal cortex during classical eyeblink conditioning using a delay conditioning paradigm with 250 ms of inter-stimulus interval. All the illustrated raster displays were collected during the 8th conditioning session (C8, 60 trials). Representative examples of three different types of firing rates as a result of the application of our spike sorting method/algorithm. The three different firing rates were characterized by having their maximum peaks close to the beginning of the CS (blue raster and firing rate, time to peak 65.3 ms, maximum rate 47.99 spikes/s); in the center of the CS-US (red raster and firing rate, time to peak 231. ms, maximum rate 38.15 spikes/s); or next to the end of the US (green raster and firing rate, time to peak 348.99 ms, maximum rate 49.59 spikes/s).
